# *Toxoplasma gondii* in the faeces of wild felids from the Atlantic Forest, Brazil

**DOI:** 10.1590/0074-02760210302

**Published:** 2022-06-27

**Authors:** Paula F Bolais, Lokman Galal, Cecília Cronemberger, Fabiane de Aguiar Pereira, Alynne da Silva Barbosa, Laís Verdan Dib, Maria Regina Reis Amendoeira, Marie-Laure Dardé, Aurélien Mercier

**Affiliations:** 1 University of Limoges, Institute of Epidemiology and Tropical Neurology, Epidemiology of Chronic Diseases in Tropical Zone, OmegaHealth, Limoges, France; 2 Instituto Chico Mendes de Conservação da Biodiversidade, Parque Nacional da Serra dos Órgãos, Teresópolis, RJ, Brasil; 3 Universidade Federal Fluminense, Instituto Biomédico, Departamento de Microbiologia e Parasitologia, Niterói, RJ, Brasil; 4 Fundação Oswaldo Cruz-Fiocruz, Instituto Oswaldo Cruz, Laboratório de Toxoplasmose e Outras Protozooses, Rio de Janeiro, RJ, Brasil; 5 Centre Hospitalier-Universitaire Dupuytren, Centre National de Référence, Toxoplasmose/Toxoplasma Biological Centre, Limoges, France

**Keywords:** Toxoplasma gondii, genetic diversity, oocyst, wild, Brazil, Felidae

## Abstract

**BACKGROUND:**

*Toxoplasma gondii* is a apicomplexan parasite of virtually all warm-blooded species. All true cats (Felidae) can act as definitive hosts for this parasite by shedding resistant oocysts into the environment. However, the patterns of oocysts shedding are only partially understood in domestic cats and largely unknown in wild felids.

**OBJECTIVES:**

We carried out molecular analysis of 82 faecal samples from wild felids collected in the Serra dos Órgãos National Park (Parnaso), Rio de Janeiro, Brazil.

**METHODS:**

We screened samples for *T. gondii* DNA using a quantitative polymerase chain reaction (qPCR) targeting the 529bp DNA fragment. Polymerase chain reaction (PCR)-positive samples were genotyped using 15 microsatellite markers.

**RESULTS:**

Only one faecal sample from a *Puma yagouaroundi* was PCR-positive [cycle threshold (Ct) = 26.88]. This sample was contaminated by a *T. gondii* strain of BrIII lineage, a common lineage in domestic animals from Brazil.

**MAIN CONCLUSIONS:**

This first report of *T. gondii* in faeces of wild South American felids in their natural environment indicates infrequent oocyst shedding and suggests a role of acquired immunity in limiting re-excretion as in domestic cats. The presence of a domestic strain of *T. gondii* in a faecal sample from a wild felid at very low concentrations (not detected by microscopy) is consistent with the hypothesis of host-parasite co-adaptations limiting the circulation of *T. gondii* strains between domestic and wild environments.

*Toxoplasma gondii* is a zoonotic protozoan having a worldwide distribution and infecting all warm-blooded species, including humans. All these species can act as intermediate hosts for *T. gondii* by developing persistent tissue-cysts after feeding from tissues of another infected intermediate host or following the ingestion of sporulated oocysts found in the environment. These oocysts are shed in the environment through the faeces of infected felids, the only definitive hosts of this parasite, and sporulate within one to five days following their excretion to become infective. Most of the studies about the role of felids in the epidemiology of *T. gondii* have focused on domestic cats. These studies shed light on several aspects of *T. gondii* epidemiology in relation to these hosts, such as the prevalence of infection, the prevalence and seasonality of oocyst shedding and the strains of *T. gondii* involved in transmission.^1^

Some noticeable aspects of oocysts shedding discovered in domestic cats are that more cats are found shedding *T. gondii* oocysts during periods (seasons) of prey abundance,^2^ a factor that may also explain the large variations in the prevalence of oocyst shedding by cats between studies.^1^ In addition, domestic cats appear to excrete oocysts for only few days following their first infection, and subsequently develop immunity to oocyst re-excretion.^3^ This explains the very low prevalence of oocysts in cat faeces, contrasting with the usual high levels of seroprevalence in cat populations.^1^ Why some cats re-excrete oocysts after developing immunity to *T. gondii* ^4^ ^,^ ^5^ is still not fully understood, although immunosuppression or concomitant infections with certain pathogens can induce re-excretion.^6^ Another interesting finding is that domestic cats do not equally spread oocysts of all *T. gondii* strains. They efficiently produce oocysts when infected with strains isolated from the domestic environment, but seldom produce oocysts when exposed to strains from the wild environment.^7^ ^,^ ^8^ This selection mechanism probably acts as an environmental barrier to the dissemination of wild strains in the domestic environment,^9^ ^,^ ^10^ in particular in continents where we still find large populations of wild felids, such as South America, and to a lesser extent North America. These wild strains have a highly divergent genetic makeup when compared to domestic strains^8^ ^,^ ^11^ ^,^ ^12^ and are nearly only found in environments where wild felids are circulating.^9^ ^,^ ^13^ ^,^ ^14^ More importantly, these strains are often highly pathogenic in immunocompetent humans while most domestic strains are known be non - or weakly - pathogenic except in some risk groups such as developing foetus in case of congenital infection and immunocompromised patients.^15^ In French Guiana (South America), unusual and severe disseminated forms of toxoplasmosis, designated as “Amazonian toxoplasmosis”, were repeatedly described in immunocompetent patients having a history of contact with the wild environment, either due to the ingestion of surface water contaminated with oocysts in the forest or the consumption of undercooked game meat, or due to the contamination of water supply with *T. gondii*.^16^ ^,^ ^17^ ^,^ ^18^ Sporadic reports of severe toxoplasmosis in immunocompetent patients are also available in other South American countries^19^ ^,^ ^20^ ^,^ ^21^ ^,^ ^22^ ^,^ ^23^ as well as in North America,^24^ ^,^ ^25^ and were also associated to contact with the wild environment. Despite the importance of wild felids in the transmission of such pathogenic strains, the epidemiology of *T. gondii* in these species is still poorly understood. Available studies have reported high levels of *T. gondii* seroprevalence in wild felids.^26^ In addition, oocyst shedding could be demonstrated in several species of wild felids,^27^ confirming their role as definitive hosts of the parasite.

As in domestic cats,^6^ ^,^ ^28^ oocyst excretion does not appear to be associated with age, since kittens of wild felids^29^ ^,^ ^30^ as well as sub-adults and adults^31^ ^,^ ^32^ ^,^ ^33^ were found to be excreting oocysts. Oocyst concentrations in wild felids were comparable to those observed in domestic cats, ranging from dozens of thousands to millions per gram of faeces.^29^ ^,^ ^32^ Wild felids seropositive for *T. gondii* appear to have lower propensity for oocyst excretion compared to seronegative ones,^34^ suggesting a role of acquired immunity in preventing oocyst excretion as in domestic cats. However, it should be kept in mind that all these data have been obtained from very few animals, and many species of felids are not represented at all in these studies. Moreover, important traits such as the frequency of oocyst shedding and host adaptation to specific parasitic strain are still poorly understood.

Here, we take advantage of a coprological survey investigating the gastrointestinal parasites of wild felids from the Serra dos Órgãos National Park (Parnaso), in the state of Rio de Janeiro, Brazil.^35^ During this survey, 82 faecal samples from wild felids were collected and investigated for the presence of different parasitic species, including *T. gondii*. Four coproparasitological techniques, followed by microscopic visualisation, were used for parasite detection. Among the 82 samples, *T. gondii*-like oocysts were detected in only one sample. However, these techniques rely on microscopic detection, which can be of limited diagnostic sensitivity. In addition, *T. gondii* oocysts cannot be visually distinguished from *Hammondia* and *Besnoitia* oocysts,^36^ limiting therefore the diagnostic specificity of this approach.

In the present study, we re-analysed these 82 samples using molecular tools to estimate the prevalence of *T. gondii* in faeces of these wild felids and to genotype the *T. gondii* strains. This study will enable a better understanding of *T. gondii* circulation in this hardly accessible wild environment and to determine which strains are disseminated by these wild felids.

## MATERIALS AND METHODS

*Ethical considerations* - The sampling protocol was approved by the Animal Ethics Committee of Fiocruz, under license number LW 53/13 and protocol number P-24/13.7, and under license number 38070, authentication code 27451648, and date of issue 19/05/2013 from the Biodiversity Information and Authorization System (SISBIO). A Convention on International Trade in Endangered Species of Wild Fauna and Flora (CITES) permit for was attributed in Brazil for the shipment of samples to Limoges, France under license number nº14BR013502/DF authorisation to export faeces of *Leopardus* spp., *Leopardus tigrinus*, *Leopardus wiedi* - Ministério do Meio Ambiente - MMA - Instituto Brasileiro do Meio Ambiente e dos Recursos Naturais Renováveis - IBAMA -CITES - License issuing system IBAMA 121480 Feline faeces.

*Faecal samples preparation* - Detailed descriptions of the study site, the sampling protocol, and the parasitological technique used for parasites detection are available elsewhere.^35^ Briefly, between March 2013 and April 2015, 3 faecal samples were collected directly from the rectum of two live felids (captured and then released in the park) and from a necropsied one. During the same period, seventy-nine cat samples were collected from the ground in the park. All these samples were stored in a refrigerator at 4ºC in plastic collectors of capacity 80 mL without chemical preservative. Four different felid species were identified through the analysis of guard hairs present in faecal samples, while felid species could not be determined for 46 samples. The 82 samples were subjected to four parasitological techniques: (i) the modified centrifuge-sedimentation technique, (ii) the centrifuge-flotation technique, (iii) the modified centrifuge-flotation technique and (iv) the spontaneous sedimentation technique. The slides obtained from these four parasitological techniques were examined and photomicrographed using an Olympus^®^ BX 41 light microscope.

*DNA extraction and real-time polymerase chain reaction (PCR)* - In July 2016, DNA was extracted from the 82 faecal samples using the NucleoSpin^®^ Soil kit (Cat. No. 74078050, Macherey-Nagel GmbH & Co.KG, Düren, Germany) as recommended in the kit manual. The extracted DNA samples were tested by a quantitative polymerase chain reaction (qPCR) assay as described by Ajzenberg et al.^37^ on a thermocycler Rotor-Gene 6000 (Corbett Life Science, Sydney, Australia), targeting the 529bp DNA fragment (REP529, GenBank accession no. AF146527^38^). In brief, each PCR contained 5 µL of extracted DNA from faeces, mixed with 15 µL of a PCR mix with 1X LightCycler FastStart DNA Master Hybridisation Probes kit (Roche diagnostics, Mannheim, Germany), 0.5 U of UDG (Roche Diagnostics, Mannheim, Germany), 5 mmol/L of MgCl2, 0.5 µmol/L of each primer, 0.1 µmol/L of TaqMan probe (Eurofins, Ebersberg, Germany) which is labeled with a fluorescent dye (6-carboxyfluorescein, 6-FAM) at 5’ end and a dark quencher (Black Hole Quencher, BHQ1) at the 3’ end. The cycling protocol was as follows: initial decontamination by UDG at 50ºC for 2 min and denaturation at 95ºC for 10 min, followed by 50 cycles at 95ºC for 20 s and 60ºC for 40 s. Each sample was run in triplicate and the results obtained were expressed in cycle threshold (Ct) values.

*Microsatellite genotyping and genetic analysis* - *T. gondii* strains were genotyped using 15 microsatellite (MS) markers distributed on 11 of the 14 chromosomes composing *T*. *gondii* genome in a single multiplex PCR-assay, as described previously.^39^ Those 15 loci included a combination of eight “typing” markers with low polymorphism (*TUB2*, *W35*, *TgM-A*, *B17*, *B18*, *M33*, *IV*.*1* and *XI*.*1*) that show little or no variation within lineages and seven “fingerprinting” markers (*M48*, *M102*, *N83*, *N82*, *AA*, *N61*, *N60*) exhibiting high polymorphism and significant variation within lineages.^40^ PCR products were sized using capillary electrophoresis on ABI PRISM 3130xl (Applied Biosystems, Foster City, CA) and the GenScan 500 ROX dye size standard (Applied Biosystems). Results were analysed using GeneMapper 5.0 software packages (Applied Biosystems).

Minimum spanning networks (MSN) based on Bruvo’s genetic distance were drawn using ‘‘Poppr” Package (implemented in R environment) to visualise the relationships between *T. gondii* strains from this study and a set of previously published strains from Brazil for comparison [Supplementary data (Table)].

## RESULTS

Only one faecal sample out of 82 samples was positive using real-time PCR (Table I) and had Ct values of 26.71; 26.90; 27.04. This positive PCR sample from a jaguarondi was not the one in which *T. gondii* oocysts were detected by Dib et al.,^35^ as this latter one (from an ocelot)was found PCR negative.

**TABLE I:** Molecular prevalence of *Toxoplasma gondii* infection in felids from the Serra dos Órgãos National Park (Parnaso), Rio de Janeiro

Species	Common name	PCR-positive/Total (%)
*Leopardus weidii*	Marguay	0/11 (0)
*Leopardus guttulus*	Southern tiger cat	0/11 (0)
*Leopardus pardalis*	Ocelot	0/5 (0)
*Puma yagouaroundi*	Jaguarondi	1/8 (12.5)
Non identified felid		0/47 (0)
Total		1/82 (1.22)

All the 15 MS markers could be successfully amplified, revealing a genotype of the Brazilian lineage BrIII, that was designated Pumayag2-RJ (Table II). The MSN showed that this genotype is closely related to other genotypes of BrIII lineage from both domestic and wild animals in Brazil (Figure).

**TABLE II:** *Toxoplasma gondii* microsatellite (MS) analysis results of the Pumayag2-RJ isolate from a jaguarondi (*Puma yagouaroundi*) and a comparison with other Brazilian Type BrIII isolates

Strain ID	Origin	Host	Type or lineage	Typing markers								Fingerprinting markers							
				TUB2	W35	TgM-A	B18	B17	M33	IV.1	XI.1	M48	M102	N60	N82	AA	N61	N83		
Pumayag2-RJ	Brazil	Jaguarondi *Puma yagouaroundi*	BrIII	289	242	205	160	348	165	278	356	213	190	142	111	263	111	312	This study
TgMytrBr SP1	Brazil	Giant anteater *Myrmecophaga tridactyla*	BrIII	289	242	205	160	348	165	278	356	213	190	142	111	263	103	312	
TgCatBr60	Brazil	Cat *Felis catis*	BrIII	289	242	205	160	348	165	278	356	213	190	142	111	263	107	312	
TgCpBr17	Brazil	Capybara *Hidrochoeris hidrochoeris*	BrIII	289	242	205	160	348	165	278	356	213	190	142	111	263	107	312	
TgCpBr36	Brazil	Capybara *Hidrochoeris hidrochoeris*	BrIII	289	242	205	160	348	165	278	356	213	190	142	111	263	107	312	
TgCkBr68	Brazil	Chicken *Gallus domesticus*	BrIII	289	242	205	160	348	165	278	356	213	190	142	111	263	123	312	
TgCatBr3	Brazil	Cat *Felis catis*	BrIII	289	242	205	160	348	165	278	356	213	190	142	111	263	113	312	
TgCkBr7	Brazil	Chicken *Gallus domesticus*	BrIII	289	242	205	160	348	165	282	356	213	190	142	111	259	103	312	
TgCpBr20	Brazil	Capybara *Hidrochoeris hidrochoeris*	BrIII	289	242	205	160	348	165	278	356	213	190	142	111	263	123	310	
TgCpBr18	Brazil	Capybara *Hidrochoeris hidrochoeris*	BrIII	289	242	205	160	348	165	278	356	213	190	142	111	261	127	310	
PS-TgRabbit BrRS1	Brazil	Rabbit *Oryctolagus cuniculus*	BrIII	291	242	205	160	348	165	278	356	213	188	142	111	261	89	314	
ENT	France	Human *Homo sapiens*	I	291	248	209	160	342	169	274	358	209	166	145	119	267	87	306	
ME49	USA	Sheep O*vis aries*	II	289	242	207	158	336	169	274	356	215	174	142	111	265	91	310	
NED	France	Human *Homo sapiens*	III	289	242	205	160	336	165	278	356	209	190	147	111	267	91	312	

References of reports describing the other Brazilian Type BrIII isolates are reported in Supplementary data (Table).

**Figure:**
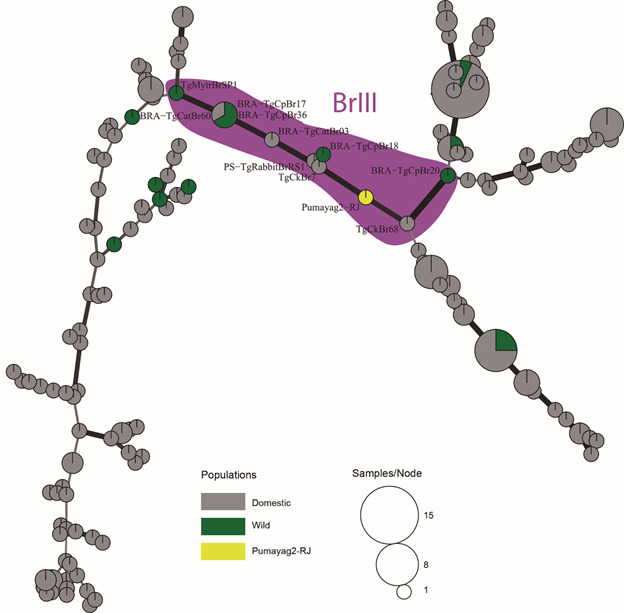
Minimum spanning network (MSN) showing the relationships between the multilocus genotype (MLG) from this study and other Brazilian MLGs from previous studies. The MSN are based on MLGs defined by 15 microsatellite markers. Each circle represents a unique MLG. The size of each circle corresponds to the number of individuals, and the colours indicate different ecotypes. Thick and dark lines show MLGs that are more closely related to each other.

## DISCUSSION

For the first time to our knowledge, we report the confirmed presence of *T. gondii* in the faeces of a wild South American felid in its natural environment. This felid was previously identified as a Jaguarondi (*Puma yagouaroundi*) by trichological analysis.^35^ It was the unique PCR-positive faecal sample among 82 tested samples.

Similarly to what is observed in domestic cats this low level of *T. gondii* prevalence in wild felid faeces should not be interpreted as an indicator of a limited circulation of *T. gondii* in this environment. In Brazil, *T. gondii* infection is highly prevalent in most regions in both domestic and wild hosts, probably due to the humid tropical climate that favours oocysts sporulation and long-term viability. The survival of oocysts also favours the contamination of intermediate hosts that are prey for felines. As an example, Furtado et al.^41^ reported a 100% seroprevalence in 31 free-ranging jaguars mainly originating from tropical areas in Brazil. In the few other reports of *T. gondii* in wild felids from other regions of the world having colder climates, seroprevalence in wild felids was more or less high, but prevalence in faeces was always markedly lower. In Finland, none of the 332 faecal samples collected on 337 Eurasian lynx (*Lynx lynx*) was positive for the presence of *T. gondii*-like oocysts, although the *T. gondii* seroprevalence in this sample was higher than 80%.^42^ In western Québec, Canada, no oocyst could be detected either in the faecal samples collected from 84 Canadian lynx (*Lynx canadensis*), although the seroprevalence of *T. gondii* in this sample was 14%.^43^ On a small sample, Aramini et al.^32^ found one out of 12 cougars (*Puma concolor*) from Vancouver Island, British Columbia, Canada, shedding *T. gondii* oocysts confirmed by mouse bioassay; eleven were seropositive. VanWormer et al.^44^ also detected *T. gondii*-like oocysts in 2 of 16 Bobcats (*Lynx rufus*) and 2 of 51 cougars from California, US, although these felids had high levels of seroprevalence: 72.7% (49.8*-*89.3) and 80.6% (69.5*-*88.9), respectively.

The pattern of oocyst excretion in wild felids is unclear. Evidence from scarce data suggests an acquired immunity to re-excretion following the first infection.^34^ Miller et al.^45^ and Jewell et al.^34^ reported short periods of oocyst shedding (5 to 10 days) in experimentally infected wild felids of different species. These shedding periods are comparable to those observed in domestic cats and are compatible with the acquisition of an immunity stopping parasitic multiplication in the cat enterocytes. In the present study, the low *T. gondii* prevalence in faeces of wild felids may also suggest a role of immunity in limiting oocysts re-excretion in wild felids as in domestic cats. However, robust estimates of oocyst shedding prevalence and knowledge of its explanatory factors (*e.g*., seasonality, prey abundance) are still lacking to conclude to a shedding prevalence comparable to the prevalence observed in domestic cats. In addition, the role of immunosuppression or concomitant infections with certain pathogens in oocyst excretion is not documented in wild felids as it is the case for domestic cats.^6^ Lukesová et al.^33^ reported three episodes of oocysts shedding (confirmed by bioassay) in a pair of captive wild cats in the Czech Republic during a period of 25 weeks (the three episodes were separated by thirteen and seven weeks, respectively), although it could not be determined which of the two cats was excreting these oocysts. In addition, they reported three episodes of oocyst shedding in a wild cat held separately during a period of 21 weeks (the three episodes were separated by six and eight weeks, respectively). These latter findings observed on a small number of individuals may suggest more frequent episodes of re-excretion than previously expected, resulting in higher frequencies of oocyst shedding in the wild. In this sense, additional well-designed studies based on long-term monitoring of captive wild felids (*e.g.*, in zoos) can be useful in understanding the patterns of oocyst re-excretion in these species.

The only PCR-positive faecal sample contained no detectable *T. gondii*-like oocysts although three different parasitological techniques were used for detection. This can be due to the lower sensitivity of microscopic detection compared to molecular detection, especially when the faecal sample contains few oocysts.^6^ It is therefore important to combine molecular techniques or mouse bioassay to microscopic detection in order to confirm the presence of *T. gondii* in faecal samples. In this sense, Salant et al.^46^ detected *T. gondii* DNA in faeces of 11 of 122 cats although oocysts could not be detected microscopically in these samples. Nevertheless, *T. gondii* DNA can be detected in the faeces of a felid following the ingestion of an infected prey without necessarily excreting oocysts.^47^ The cysts in the tissues of the prey are digested in the cat’s gut and traces of DNA from bradyzoites are then detected in the cat’s faeces. However, experimental evidence shows that DNA from bradyzoites can only be retrieved in tiny amounts in faeces (Ct higher than 35). Here, the concentration of *T. gondii* DNA found in the positive sample from this study was much higher (Ct = 26.88). This makes it unlikely that the detected DNA is only the digestion product of tissue-cysts from an infected prey. This finding shows that the concentration of *T. gondii* DNA in the sample could provide crucial indication to differentiate between presence of oocysts and traces of DNA from digested bradyzoites, although it does not allow confirmation for the presence of oocysts. In this sense, supplementing the diagnosis with mouse bioassay would be useful to ascertain the presence of oocysts in the faecal sample although it raises ethical concerns that should be taken in consideration. In this case, the storage conditions of the samples must be taken into account due to the progressive decrease of oocysts viability with time.^48^ Overall, our results show that few oocysts can be excreted in certain situations. The use of microscopic detection only in many previous studies has probably led to an underestimation of oocyst shedding and only faecal samples containing hundreds of thousands or millions of oocysts could be identified.

At the opposite, the faecal sample from an ocelot in which Dib et al.^35^ observed *T. gondii*-like oocysts was found PCR-negative. This case also shows the importance of confirming the presence of *T. gondii* by either molecular techniques or mouse bioassay. These *T. gondii*-like oocysts are probably of *Hammondia* spp., which occur quite as often as *T. gondii* in cat faeces, but less common coccidian species such *Besnoitia* spp. or other undescribed species could be occurring. As an example, Herrmann et al.^28^ collected 18,259 faecal samples from cats in Germany: *T. gondii*-like oocysts were detected in 105 samples; only 46 were PCR positive, 34 turned out to be *H. hammondi* and 25 were PCR negative to *T. gondii*, *Neospora caninum* and *Besnoitia besnoiti*. In the same way, VanWormer et al.^44^ could confirm the presence of *T. gondii* by PCR for only two of the four oocyst-positive samples from wild Californian felids. It is therefore likely that coprological surveys relying only on microscopic detection^49^ ^,^ ^50^ have overestimated the prevalence of *T. gondii* in faecal samples.

A *T. gondii* strain of the BrIII lineage was identified in the PCR positive faecal sample from this study; this common Brazilian lineage (the second most common Brazilian clonal lineage) has been repeatedly isolated from tissue samples of domestic and wild animals in the country.^51^ ^,^ ^52^ ^,^ ^53^ The previous isolation of this lineage from domestic cats and intermediate hosts (chickens, rabbits) suggest that this lineage is well adapted to the domestic environment, and it is likely that domestic cats efficiently disseminated it in the form of oocysts. It shares 7 of 8 typing alleles with type III lineage (348 instead of 336 at B17 marker), a very common world-wide lineage that is well adapted to transmission by domestic cats.^54^ The circulation of BrIII lineage in wild animals (isolated from capybaras and from a giant anteater) could be due to the proximity between wildlife and domestic environment observed in many regions of Brazil^55^ ^,^ ^56^ as in our study with the proximity between Serra dos Órgãos National Park and Rio de Janeiro (50 km). The genotypes of this lineage from domestic and wild animals were closely intermingled in the MSN, a pattern supporting an active circulation of *T. gondii* between wild and domestic environments in Brazil. Increasing anthropogenic pressure over natural habitats results in fragmentation of the landscape and wild animals are often in close contact with human settlements. Unfortunately, the Atlantic Forest, biome where is located the Serra dos Órgãos National Park, is not an exception to this undergoing process.^57^ A different situation has been described in French Guiana where domestic and wild *T. gondii* populations were highly divergent,^9^ probably due to the relatively well-preserved wild environment in this area.^58^ The PCR-positive faecal sample from this study was collected in a popular hiking trail, about three kilometres away from the closest human settlement. In these new human settlements, domestic cats proliferate, resulting in the contamination of nearby soils and water streams with oocysts. In this situation, wild felids can then get infected by predating on domestic animals or on wild animals previously exposed to domestic *T. gondii* strains. Domestic cats have been registered by camera traps in some trails of Parnaso, particularly those closer to human settlements (Cronemberger, unpublished data). In this scenario, it is important to raise awareness among the general population, but especially among the neighbourhood of natural areas, about the importance of responsible ownership of domestic animals, with adequate vaccination, health care and castration, to reduce the chances of transmission of diseases between domestic and wild animals.

It is unclear whether BrIII lineage - and other lineages commonly found in the domestic environment - are efficiently spread by wild felids. Jewell et al.^34^ and Miller et al.^45^ conducted experimental infections of wild felids using a domestic *T. gondii* strain named M-7741 (type III strain from a sheep in the USA). Only one out of six seronegative bobcats (*Lynx rufus*) excreted oocysts after being challenged with M-7741. None of the two seronegative margay cats (*Leopardus wiedii*) challenged with the same strain excreted oocysts. However, one seronegative cougar (*Puma concolor*) and one seronegative Jaguarondi (*Puma yagouaroundi*) challenged with this strain excreted oocysts. Interestingly, two Asian leopard cats (*Prionailurus bengalensis*) excreted oocysts after being challenged with LRH strain, but not after a previous challenge with M-7741 strain. LRH strain was previously isolated from the faeces of a Panamanian ocelot (*Felis pardalis*), and is therefore probably a true wild strain.^34^ Overall, it is for now impossible to demonstrate clear species-specific patterns based on these small samples. At the opposite, Khan et al.^8^ challenged two groups of domestic cats (eight and six cats) with four domestic and three wild *T. gondii* strains, respectively (one strain for two cats). The four domestic strains caused oocyst excretion in six of the eight exposed cats (two strains caused oocyst excretion in only one of the two exposed cats) while the three wild strains were associated with oocyst excretion in only one of the six cats exposed. These findings suggest the existence of host-parasite co-adaptations that are probably limiting the circulation of *T. gondii* strains between domestic and wild environments, although these specific adaptations show some degree of permissiveness for oocyst shedding by felids from a different environment. In this study, the presence of a domestic lineage of *T. gondii* (BrIII) in a faecal sample from a wild felid at very low concentrations (not detected by microscopy) could be consistent with the hypothesis of a poor adaptation of felids to *T. gondii* strains from a different environment. More studies are needed to characterise these putative host-parasite co-adaptations.

In conclusion, our findings highlight the difficulties of studying patterns of oocysts excretion in felids and the importance of using more than one diagnostic technique to verify for the presence of oocysts in a faecal sample. We provide additional evidence for the diffusion of domestic strains to wildlife in Brazil, probably due to the increasing anthropogenic pressure over natural habitats in this area of the world. In this context, wild species are likely to be exposed to new *T. gondii* strains from the domestic environment. There is well-documented evidence for an influence of *T. gondii* genotype on disease outcome in humans^10^ ^,^ ^16^ ^,^ ^17^ ^,^ ^18^ and in mice,^59^ ^,^ ^60^ but studies in wildlife populations are scarce.^61^ These exposures to new strains could have an underestimated impact on wildlife and deserve further investigations.
