# Supplementary material for: Toxoplasma gondii in the faeces of wild felids from the Atlantic Forest, Brazil
Source: Mem Inst Oswaldo Cruz. 2022 Jun 27;117:e210302. doi: 10.1590/0074-02760210302 (PMC9241165; doi:10.1590/0074-02760210302)
Supplement: Supplementary file 1 [file 1678-8060-mioc-117-e210302-s.pdf]

Toxoplasma gondii genotyping results with 15 microsatellite markers of strains from Brazil

			Microsatellite marker															
	Origin	Species	*TUB2*	*W35*	*TgM-A*	*B18*	*B17*	*M33*	*MIV.1*	*MXI.1*	*M48*	*M102*	*N60*	*N82*	*AA*	*N61*	*N83*	References
BRA-Cat1FN	Fernando de Noronha	Cat (*Felis catis*)	289	242	207	158	336	169	274	356	245	178	138	123	271	95	329	Silva, Jean Carlos Ramos, et al. "Cat-rodent Toxoplasma gondii Type II-variant circulation and limited genetic diversity on the Island of Fernando de Noronha, Brazil." Parasites & vectors 10.1 (2017): 1-6.
BRA-PBR	São Paulo	Dog (*Canis familiaris*)	291	248	205	160	334	165	274	354	213	190	140	119	271	87	308	Su, Chunlei, et al. "Globally diverse Toxoplasma gondii isolates comprise six major clades originating from a small number of distinct ancestral lineages." Proceedings of the National Academy of Sciences 109.15 (2012): 5844-5849.
BRA-TgBatBr01	São Paulo	Velvety free-tailed bat (*Molossus molossus*)	291	242	205	160	362	165	278	356	229	174	153	111	265	91	314	Cabral AD, et al. First isolation and genotyping of Toxoplasma gondii from bats (Mammalia: Chiroptera). Vet Parasitol. 2013 Mar 31;193(1-3):100-4.
BRA-TgBatBr02	São Paulo	Common vampire bat (*Desmodus rotundus*)	291	242	205	160	362	165	278	356	229	174	140	111	265	91	314	Cabral AD, et al. First isolation and genotyping of Toxoplasma gondii from bats (Mammalia: Chiroptera). Vet Parasitol. 2013 Mar 31;193(1-3):100-4.
BRA-TgCatBr03	São Paulo	Cat (*Felis catis*)	289	242	205	160	348	165	278	356	213	190	142	111	263	113	312	Su, Chunlei, et al. "Globally diverse Toxoplasma gondii isolates comprise six major clades originating from a small number of distinct ancestral lineages." Proceedings of the National Academy of Sciences 109.15 (2012): 5844-5849.
BRA-TgCatBr05	São Paulo	Cat (*Felis catis*)	291	242	205	160	362	165	278	356	237	174	140	111	265	89	314	Su, Chunlei, et al. "Globally diverse Toxoplasma gondii isolates comprise six major clades originating from a small number of distinct ancestral lineages." Proceedings of the National Academy of Sciences 109.15 (2012): 5844-5849.
BRA-TgCatBr06	São Paulo	Cat (*Felis catis*)	291	242	205	162	342	169	272	356	237	164	145	111	265	89	314	Su, Chunlei, et al. "Globally diverse Toxoplasma gondii isolates comprise six major clades originating from a small number of distinct ancestral lineages." Proceedings of the National Academy of Sciences 109.15 (2012): 5844-5849.
BRA-TgCatBr09	São Paulo	Cat (*Felis catis*)	291	242	205	160	362	165	278	354	227	174	140	111	269	89	308	Su, Chunlei, et al. "Globally diverse Toxoplasma gondii isolates comprise six major clades originating from a small number of distinct ancestral lineages." Proceedings of the National Academy of Sciences 109.15 (2012): 5844-5849.
BRA-TgCatBr10	São Paulo	Cat (*Felis catis*)	291	242	207	160	360	165	278	356	229	174	140	105	263	91	314	Su, Chunlei, et al. "Globally diverse Toxoplasma gondii isolates comprise six major clades originating from a small number of distinct ancestral lineages." Proceedings of the National Academy of Sciences 109.15 (2012): 5844-5849.
BRA-TgCatBr15	São Paulo	Cat (*Felis catis*)	289	242	205	162	344	165	278	358	225	164	142	111	263	105	312	Su, Chunlei, et al. "Globally diverse Toxoplasma gondii isolates comprise six major clades originating from a small number of distinct ancestral lineages." Proceedings of the National Academy of Sciences 109.15 (2012): 5844-5849.
BRA-TgCatBr18	São Paulo	Cat (*Felis catis*)	291	242	207	160	338	169	272	358	229	164	142	111	263	89	308	Su, Chunlei, et al. "Globally diverse Toxoplasma gondii isolates comprise six major clades originating from a small number of distinct ancestral lineages." Proceedings of the National Academy of Sciences 109.15 (2012): 5844-5849.
BRA-TgCatBr20	São Paulo	Cat (*Felis catis*)	289	242	205	160	360	165	278	356	213	174	140	105	265	105	314	Su, Chunlei, et al. "Globally diverse Toxoplasma gondii isolates comprise six major clades originating from a small number of distinct ancestral lineages." Proceedings of the National Academy of Sciences 109.15 (2012): 5844-5849.
BRA-TgCatBr25	São Paulo	Cat (*Felis catis*)	291	242	207	160	338	169	272	358	229	164	142	111	263	89	308	Su, Chunlei, et al. "Globally diverse Toxoplasma gondii isolates comprise six major clades originating from a small number of distinct ancestral lineages." Proceedings of the National Academy of Sciences 109.15 (2012): 5844-5849.
BRA-TgCatBr26	São Paulo	Cat (*Felis catis*)	291	248	205	160	362	165	278	354	229	174	140	111	271	89	308	Su, Chunlei, et al. "Globally diverse Toxoplasma gondii isolates comprise six major clades originating from a small number of distinct ancestral lineages." Proceedings of the National Academy of Sciences 109.15 (2012): 5844-5849.
BRA-TgCatBr34	São Paulo	Cat (*Felis catis*)	291	248	205	160	338	169	272	356	245	164	136	111	316	87	314	Su, Chunlei, et al. "Globally diverse Toxoplasma gondii isolates comprise six major clades originating from a small number of distinct ancestral lineages." Proceedings of the National Academy of Sciences 109.15 (2012): 5844-5849.
BRA-TgCatBr38	São Paulo	Cat (*Felis catis*)	291	242	205	162	362	165	278	356	221	166	149	111	261	93	337	Pena HF et al. Population structure and mouse-virulence of Toxoplasma gondii in Brazil. Int J Parasitol. 2008 Apr;38(5):561-9.
BRA-TgCatBr39	São Paulo	Cat (*Felis catis*)	289	242	205	160	342	165	278	358	233	164	145	111	316	89	308	Pena HF et al. Population structure and mouse-virulence of Toxoplasma gondii in Brazil. Int J Parasitol. 2008 Apr;38(5):561-9.
BRA-TgCatBr40	São Paulo	Cat (*Felis catis*)	291	242	207	162	338	169	272	358	223	176	145	113	316	91	308	Pena HF et al. Population structure and mouse-virulence of Toxoplasma gondii in Brazil. Int J Parasitol. 2008 Apr;38(5):561-9.
BRA-TgCatBr41	São Paulo	Cat (*Felis catis*)	291	242	205	160	342	165	278	354	231	166	149	111	263	91	308	Pena HF et al. Population structure and mouse-virulence of Toxoplasma gondii in Brazil. Int J Parasitol. 2008 Apr;38(5):561-9.
BRA-TgCatBr42	São Paulo	Cat (*Felis catis*)	291	248	205	160	342	165	274	354	229	166	147	111	273	89	308	Pena HF et al. Population structure and mouse-virulence of Toxoplasma gondii in Brazil. Int J Parasitol. 2008 Apr;38(5):561-9.
BRA-TgCatBr44	São Paulo	Cat (*Felis catis*)	291	242	205	162	342	165	278	356	231	166	153	111	265	91	345	Pena HF et al. Population structure and mouse-virulence of Toxoplasma gondii in Brazil. Int J Parasitol. 2008 Apr;38(5):561-9.
BRA-TgCatBr45	São Paulo	Cat (*Felis catis*)	291	242	205	160	348	169	272	354	237	166	147	111	269	89	306	Pena HF et al. Population structure and mouse-virulence of Toxoplasma gondii in Brazil. Int J Parasitol. 2008 Apr;38(5):561-9.
BRA-TgCatBr50	São Paulo	Cat (*Felis catis*)	291	248	205	160	342	165	274	354	231	166	147	111	265	89	310	Pena HF et al. Population structure and mouse-virulence of Toxoplasma gondii in Brazil. Int J Parasitol. 2008 Apr;38(5):561-9.
BRA-TgCatBr52	São Paulo	Cat (*Felis catis*)	289	242	205	160	342	165	278	358	231	164	145	111	316	89	308	Pena HF et al. Population structure and mouse-virulence of Toxoplasma gondii in Brazil. Int J Parasitol. 2008 Apr;38(5):561-9.
BRA-TgCatBr55	São Paulo	Cat (*Felis catis*)	291	248	207	160	342	169	272	356	249	166	145	111	267	89	312	Pena HF et al. Population structure and mouse-virulence of Toxoplasma gondii in Brazil. Int J Parasitol. 2008 Apr;38(5):561-9.
BRA-TgCatBr57	São Paulo	Cat (*Felis catis*)	289	242	203	164	338	169	272	356	219	180	142	105	289	81	333	Pena HF et al. Population structure and mouse-virulence of Toxoplasma gondii in Brazil. Int J Parasitol. 2008 Apr;38(5):561-9.
BRA-TgCatBr60	São Paulo	Cat (*Felis catis*)	289	242	205	160	348	165	278	356	213	190	142	111	263	107	312	Pena HF et al. Population structure and mouse-virulence of Toxoplasma gondii in Brazil. Int J Parasitol. 2008 Apr;38(5):561-9.

	Origin	Species	Microsatellite marker															References
			TUB2	*W35*	*TgM-A*	*B18*	*B17*	*M33*	*MIV.1*	*MXI.1*	*M48*	*M102*	*N60*	*N82*	*AA*	*N61*	*N83*	
BRA-TgCatBr62	São Paulo	Cat (*Felis catis*)	291	248	205	160	342	165	274	354	225	166	145	111	265	89	310	Pena HF et al. Population structure and mouse-virulence of Toxoplasma gondii in Brazil. Int J Parasitol. 2008 Apr;38(5):561-9.
BRA-TgCatBr64	São Paulo	Cat (*Felis catis*)	289	242	207	160	338	165	278	356	225	190	136	105	263	97	310	Pena HF et al. Population structure and mouse-virulence of Toxoplasma gondii in Brazil. Int J Parasitol. 2008 Apr;38(5):561-9.
BRA-TgCatBr66	São Paulo	Cat (*Felis catis*)	289	248	209	160	336	165	278	356	211	190	147	111	267	89	306	Pena HF et al. Population structure and mouse-virulence of Toxoplasma gondii in Brazil. Int J Parasitol. 2008 Apr;38(5):561-9.
BRA-TgCatBr67	São Paulo	Cat (*Felis catis*)	289	242	205	160	348	165	278	358	213	164	145	111	263	123	308	Pena HF et al. Population structure and mouse-virulence of Toxoplasma gondii in Brazil. Int J Parasitol. 2008 Apr;38(5):561-9.
BRA-TgCatBr68	São Paulo	Cat (*Felis catis*)	289	242	205	160	342	165	278	358	239	164	145	111	316	89	308	Pena HF et al. Population structure and mouse-virulence of Toxoplasma gondii in Brazil. Int J Parasitol. 2008 Apr;38(5):561-9.
BRA-TgCatBr69	São Paulo	Cat (*Felis catis*)	291	242	205	162	342	165	278	356	233	166	166	111	265	91	341	Pena HF et al. Population structure and mouse-virulence of Toxoplasma gondii in Brazil. Int J Parasitol. 2008 Apr;38(5):561-9.
BRA-TgCatBr71	São Paulo	Cat (*Felis catis*)	291	248	205	160	342	165	274	354	233	166	149	111	271	89	308	Pena HF et al. Population structure and mouse-virulence of Toxoplasma gondii in Brazil. Int J Parasitol. 2008 Apr;38(5):561-9.
BRA-TgCatBr72	São Paulo	Cat (*Felis catis*)	289	248	205	160	342	165	274	354	231	164	147	111	271	89	308	Pena HF et al. Population structure and mouse-virulence of Toxoplasma gondii in Brazil. Int J Parasitol. 2008 Apr;38(5):561-9.
BRA-TgCatBr75	São Paulo	Cat (*Felis catis*)	291	248	205	160	342	165	274	354	235	166	147	111	271	87	306	Pena HF et al. Population structure and mouse-virulence of Toxoplasma gondii in Brazil. Int J Parasitol. 2008 Apr;38(5):561-9.
BRA-TgCatBr76	São Paulo	Cat (*Felis catis*)	291	242	205	160	342	165	278	356	237	176	140	111	265	93	314	Pena HF et al. Population structure and mouse-virulence of Toxoplasma gondii in Brazil. Int J Parasitol. 2008 Apr;38(5):561-9.
BRA-TgCatBr78	São Paulo	Cat (*Felis catis*)	289	242	205	160	342	165	278	358	231	164	145	111	316	89	308	Pena HF et al. Population structure and mouse-virulence of Toxoplasma gondii in Brazil. Int J Parasitol. 2008 Apr;38(5):561-9.
BRA-TgCatBr80	São Paulo	Cat (*Felis catis*)	289	248	209	160	342	169	272	356	233	166	142	123	265	87	306	Pena HF et al. Population structure and mouse-virulence of Toxoplasma gondii in Brazil. Int J Parasitol. 2008 Apr;38(5):561-9.
BRA-TgCatBr81	São Paulo	Cat (*Felis catis*)	291	242	207	156	338	165	276	358	225	166	145	107	265	91	316	Pena HF et al. Population structure and mouse-virulence of Toxoplasma gondii in Brazil. Int J Parasitol. 2008 Apr;38(5):561-9.
BRA-TgCkBr008	São Paulo	Chicken (*Gallus domesticus*)	289	242	203	156	342	169	278	358	229	170	147	105	265	91	312	Su, Chunlei, et al. "Globally diverse Toxoplasma gondii isolates comprise six major clades originating from a small number of distinct ancestral lineages." Proceedings of the National Academy of Sciences 109.15 (2012): 5844-5849.
BRA-TgCkBr013	São Paulo	Chicken (*Gallus domesticus*)	289	242	205	160	338	169	276	356	213	164	145	111	316	89	312	Su, Chunlei, et al. "Globally diverse Toxoplasma gondii isolates comprise six major clades originating from a small number of distinct ancestral lineages." Proceedings of the National Academy of Sciences 109.15 (2012): 5844-5849.
BRA-TgCkBr016	São Paulo	Chicken (*Gallus domesticus*)	291	242	205	160	362	165	278	356	229	164	136	113	265	91	314	Su, Chunlei, et al. "Globally diverse Toxoplasma gondii isolates comprise six major clades originating from a small number of distinct ancestral lineages." Proceedings of the National Academy of Sciences 109.15 (2012): 5844-5849.
BRA-TgCkBr019	São Paulo	Chicken (*Gallus domesticus*)	289	242	207	160	342	165	278	358	223	164	136	111	318	87	314	Su, Chunlei, et al. "Globally diverse Toxoplasma gondii isolates comprise six major clades originating from a small number of distinct ancestral lineages." Proceedings of the National Academy of Sciences 109.15 (2012): 5844-5849.
BRA-TgCkBr026	Rio de Janeiro	Chicken (*Gallus domesticus*)	291	242	207	162	342	165	278	358	229	164	151	105	265	91	310	Su, Chunlei, et al. "Globally diverse Toxoplasma gondii isolates comprise six major clades originating from a small number of distinct ancestral lineages." Proceedings of the National Academy of Sciences 109.15 (2012): 5844-5849.
BRA-TgCkBr036	Rio de Janeiro	Chicken (*Gallus domesticus*)	291	246	207	162	358	169	276	358	221	164	142	105	263	91	314	Su, Chunlei, et al. "Globally diverse Toxoplasma gondii isolates comprise six major clades originating from a small number of distinct ancestral lineages." Proceedings of the National Academy of Sciences 109.15 (2012): 5844-5849.
BRA-TgCkBr037	Rio de Janeiro	Chicken (*Gallus domesticus*)	291	242	205	162	338	165	276	356	231	164	138	105	289	105	331	Su, Chunlei, et al. "Globally diverse Toxoplasma gondii isolates comprise six major clades originating from a small number of distinct ancestral lineages." Proceedings of the National Academy of Sciences 109.15 (2012): 5844-5849.
BRA-TgCkBr038	Rio de Janeiro	Chicken (*Gallus domesticus*)	291	246	207	162	362	169	272	358	233	164	142	105	322	87	314	Su, Chunlei, et al. "Globally diverse Toxoplasma gondii isolates comprise six major clades originating from a small number of distinct ancestral lineages." Proceedings of the National Academy of Sciences 109.15 (2012): 5844-5849.
BRA-TgCkBr040	Rio de Janeiro	Chicken (*Gallus domesticus*)	291	248	205	160	342	165	274	356	235	166	147	111	269	89	308	Su, Chunlei, et al. "Globally diverse Toxoplasma gondii isolates comprise six major clades originating from a small number of distinct ancestral lineages." Proceedings of the National Academy of Sciences 109.15 (2012): 5844-5849.
BRA-TgCkBr041	Rio de Janeiro	Chicken (*Gallus domesticus*)	291	248	207	162	366	169	272	358	233	164	142	111	269	91	314	Su, Chunlei, et al. "Globally diverse Toxoplasma gondii isolates comprise six major clades originating from a small number of distinct ancestral lineages." Proceedings of the National Academy of Sciences 109.15 (2012): 5844-5849.
BRA-TgCkBr045	Rio de Janeiro	Chicken (*Gallus domesticus*)	291	242	207	164	342	169	272	356	229	172	142	105	320	81	310	Su, Chunlei, et al. "Globally diverse Toxoplasma gondii isolates comprise six major clades originating from a small number of distinct ancestral lineages." Proceedings of the National Academy of Sciences 109.15 (2012): 5844-5849.
BRA-TgCkBr048	Rio de Janeiro	Chicken (*Gallus domesticus*)	289	242	205	164	362	165	278	356	225	164	157	111	297	91	343	Su, Chunlei, et al. "Globally diverse Toxoplasma gondii isolates comprise six major clades originating from a small number of distinct ancestral lineages." Proceedings of the National Academy of Sciences 109.15 (2012): 5844-5849.
BRA-TgCkBr054	Rio de Janeiro	Chicken (*Gallus domesticus*)	291	248	203	160	342	165	274	354	221	166	142	111	269	89	329	Su, Chunlei, et al. "Globally diverse Toxoplasma gondii isolates comprise six major clades originating from a small number of distinct ancestral lineages." Proceedings of the National Academy of Sciences 109.15 (2012): 5844-5849.
BRA-TgCkBr059	Rio de Janeiro	Chicken (*Gallus domesticus*)	291	248	205	160	342	165	278	354	227	166	145	105	261	93	306	Su, Chunlei, et al. "Globally diverse Toxoplasma gondii isolates comprise six major clades originating from a small number of distinct ancestral lineages." Proceedings of the National Academy of Sciences 109.15 (2012): 5844-5849.

	Origin	Species	Microsatellite marker															References
			TUB2	*W35*	*TgM-A*	*B18*	*B17*	*M33*	*MIV.1*	*MXI.1*	*M48*	*M102*	*N60*	*N82*	*AA*	*N61*	*N83*	
BRA-TgCkBr061	Rio de Janeiro	Chicken (*Gallus domesticus*)	291	242	205	162	338	165	276	356	235	176	140	119	289	99	331	Su, Chunlei, et al. “Globally diverse Toxoplasma gondii isolates comprise six major clades originating from a small number of distinct ancestral lineages.” Proceedings of the National Academy of Sciences 109.15 (2012): 5844-5849.
BRA-TgCkBr074	Rio de Janeiro	Chicken (*Gallus domesticus*)	291	242	207	162	340	169	272	358	227	164	138	111	263	91	314	Su, Chunlei, et al. “Globally diverse Toxoplasma gondii isolates comprise six major clades originating from a small number of distinct ancestral lineages.” Proceedings of the National Academy of Sciences 109.15 (2012): 5844-5849.
BRA-TgCkBr075	Rio de Janeiro	Chicken (*Gallus domesticus*)	291	242	207	162	342	165	278	358	231	164	155	107	295	95	308	Su, Chunlei, et al. “Globally diverse Toxoplasma gondii isolates comprise six major clades originating from a small number of distinct ancestral lineages.” Proceedings of the National Academy of Sciences 109.15 (2012): 5844-5849.
BRA-TgCkBr089	Rio de Janeiro	Chicken (*Gallus domesticus*)	291	246	207	162	362	169	278	358	227	164	142	107	326	87	314	Su, Chunlei, et al. “Globally diverse Toxoplasma gondii isolates comprise six major clades originating from a small number of distinct ancestral lineages.” Proceedings of the National Academy of Sciences 109.15 (2012): 5844-5849.
BRA-TgCkBr093	Paraná	Chicken (*Gallus domesticus*)	291	242	207	160	342	165	278	358	235	174	140	111	265	91	308	Su, Chunlei, et al. “Globally diverse Toxoplasma gondii isolates comprise six major clades originating from a small number of distinct ancestral lineages.” Proceedings of the National Academy of Sciences 109.15 (2012): 5844-5849.
BRA-TgCkBr107	Pará	Chicken (*Gallus domesticus*)	289	242	205	162	340	165	278	358	243	164	145	111	267	93	306	Su, Chunlei, et al. “Globally diverse Toxoplasma gondii isolates comprise six major clades originating from a small number of distinct ancestral lineages.” Proceedings of the National Academy of Sciences 109.15 (2012): 5844-5849.
BRA-TgCkBr109	Pará	Chicken (*Gallus domesticus*)	291	246	203	156	336	167	276	356	215	174	145	111	265	101	316	Su, Chunlei, et al. “Globally diverse Toxoplasma gondii isolates comprise six major clades originating from a small number of distinct ancestral lineages.” Proceedings of the National Academy of Sciences 109.15 (2012): 5844-5849.
BRA-TgCkBr126	Rondônia	Chicken (*Gallus domesticus*)	289	248	205	160	342	169	278	354	213	166	142	111	269	105	310	Su, Chunlei, et al. “Globally diverse Toxoplasma gondii isolates comprise six major clades originating from a small number of distinct ancestral lineages.” Proceedings of the National Academy of Sciences 109.15 (2012): 5844-5849.
BRA-TgCkBr130	Rondônia	Chicken (*Gallus domesticus*)	289	242	205	160	348	169	278	356	213	192	142	111	265	103	314	Su, Chunlei, et al. “Globally diverse Toxoplasma gondii isolates comprise six major clades originating from a small number of distinct ancestral lineages.” Proceedings of the National Academy of Sciences 109.15 (2012): 5844-5849.
BRA-TgCkBr136	Rondônia	Chicken (*Gallus domesticus*)	291	242	205	160	342	165	278	354	229	166	140	111	271	91	308	Su, Chunlei, et al. “Globally diverse Toxoplasma gondii isolates comprise six major clades originating from a small number of distinct ancestral lineages.” Proceedings of the National Academy of Sciences 109.15 (2012): 5844-5849.
BRA-TgCkBr141	Pará	Chicken (*Gallus domesticus*)	291	248	209	160	344	165	278	356	209	166	142	113	281	95	306	Su, Chunlei, et al. “Globally diverse Toxoplasma gondii isolates comprise six major clades originating from a small number of distinct ancestral lineages.” Proceedings of the National Academy of Sciences 109.15 (2012): 5844-5849.
BRA-TgCkBr143	Pará	Chicken (*Gallus domesticus*)	289	242	205	162	340	169	278	358	237	164	147	111	293	95	308	Su, Chunlei, et al. “Globally diverse Toxoplasma gondii isolates comprise six major clades originating from a small number of distinct ancestral lineages.” Proceedings of the National Academy of Sciences 109.15 (2012): 5844-5849.
BRA-TgCkBr147	Rio Grande do Sul	Chicken (*Gallus domesticus*)	289	242	205	162	334	169	272	356	227	164	142	117	334	87	308	Su, Chunlei, et al. “Globally diverse Toxoplasma gondii isolates comprise six major clades originating from a small number of distinct ancestral lineages.” Proceedings of the National Academy of Sciences 109.15 (2012): 5844-5849.
BRA-TgCkBr155	Rio Grande do Sul	Chicken (*Gallus domesticus*)	289	242	205	160	334	167	276	356	213	190	140	119	267	87	308	Su, Chunlei, et al. “Globally diverse Toxoplasma gondii isolates comprise six major clades originating from a small number of distinct ancestral lineages.” Proceedings of the National Academy of Sciences 109.15 (2012): 5844-5849.
BRA-TgCkBr165	Pernambuco	Chicken (*Gallus domesticus*)	291	242	205	162	342	165	278	356	213	164	142	109	279	87	312	Silva, Jean Carlos Ramos, et al. “Cat-rodent Toxoplasma gondii Type II-variant circulation and limited genetic diversity on the Island of Fernando de Noronha, Brazil.” Parasites & vectors 10.1 (2017): 1-6.
BRA-TgCkBr166	Pernambuco	Chicken (*Gallus domesticus*)	289	242	207	160	342	165	278	356	211	190	149	105	263	101	306	Silva, Jean Carlos Ramos, et al. “Cat-rodent Toxoplasma gondii Type II-variant circulation and limited genetic diversity on the Island of Fernando de Noronha, Brazil.” Parasites & vectors 10.1 (2017): 1-6.
BRA-TgCkBr168	Northeast Brazil	Chicken (*Gallus domesticus*)	291	242	207	158	336	169	274	356	235	176	138	119	261	93	310	Silva, Jean Carlos Ramos, et al. “Cat-rodent Toxoplasma gondii Type II-variant circulation and limited genetic diversity on the Island of Fernando de Noronha, Brazil.” Parasites & vectors 10.1 (2017): 1-6.
BRA-TgCkBr169	Northeast Brazil	Chicken (*Gallus domesticus*)	291	242	205	162	342	165	278	356	227	164	142	109	277	91	312	Silva, Jean Carlos Ramos, et al. “Cat-rodent Toxoplasma gondii Type II-variant circulation and limited genetic diversity on the Island of Fernando de Noronha, Brazil.” Parasites & vectors 10.1 (2017): 1-6.
BRA-TgCkBr171	Northeast Brazil	Chicken (*Gallus domesticus*)	289	242	207	160	348	169	278	356	213	196	147	111	265	87	308	Silva, Jean Carlos Ramos, et al. “Cat-rodent Toxoplasma gondii Type II-variant circulation and limited genetic diversity on the Island of Fernando de Noronha, Brazil.” Parasites & vectors 10.1 (2017): 1-6.
BRA-TgCkBr173	Northeast Brazil	Chicken (*Gallus domesticus*)	289	248	205	160	348	169	278	354	213	194	149	111	275	89	308	Silva, Jean Carlos Ramos, et al. “Cat-rodent Toxoplasma gondii Type II-variant circulation and limited genetic diversity on the Island of Fernando de Noronha, Brazil.” Parasites & vectors 10.1 (2017): 1-6.
BRA-TgCkBr177	Northeast Brazil	Chicken (*Gallus domesticus*)	291	242	207	166	334	165	278	356	239	168	147	105	NA	93	310	Silva, Jean Carlos Ramos, et al. “Cat-rodent Toxoplasma gondii Type II-variant circulation and limited genetic diversity on the Island of Fernando de Noronha, Brazil.” Parasites & vectors 10.1 (2017): 1-6.
BRA-TgCkBr178	Northeast Brazil	Chicken (*Gallus domesticus*)	291	242	205	160	336	165	278	356	213	NA	142	109	299	101	312	Su, Chunlei, et al. “Globally diverse Toxoplasma gondii isolates comprise six major clades originating from a small number of distinct ancestral lineages.” Proceedings of the National Academy of Sciences 109.15 (2012): 5844-5849.
BRA-TgCkBr186	Northeast Brazil	Chicken (*Gallus domesticus*)	291	248	205	160	342	165	278	354	237	166	142	105	263	89	306	Silva, Jean Carlos Ramos, et al. “Cat-rodent Toxoplasma gondii Type II-variant circulation and limited genetic diversity on the Island of Fernando de Noronha, Brazil.” Parasites & vectors 10.1 (2017): 1-6.
BRA-TgCkBr210	Fernando de Noronha	Chicken (*Gallus domesticus*)	289	242	209	160	336	165	278	356	213	190	145	111	267	87	308	Silva, Jean Carlos Ramos, et al. “Cat-rodent Toxoplasma gondii Type II-variant circulation and limited genetic diversity on the Island of Fernando de Noronha, Brazil.” Parasites & vectors 10.1 (2017): 1-6.

			Microsatellite marker															
	Origin	Species	*TUB2*	*W35*	*TgM-A*	*B18*	*B17*	*M33*	*MIV.1*	*MXI.1*	*M48*	*M102*	*N60*	*N82*	*AA*	*N61*	*N83*	References
BRA-TgCkBr211	Fernando de Noronha	Chicken (*Gallus domesticus*)	289	242	209	160	336	165	278	356	213	190	145	111	265	87	308	Silva, Jean Carlos Ramos, et al. “Cat-rodent Toxoplasma gondii Type II-variant circulation and limited genetic diversity on the Island of Fernando de Noronha, Brazil.” Parasites & vectors 10.1 (2017): 1-6.
BRA-TgCkBr212	Fernando de Noronha	Chicken (*Gallus domesticus*)	289	242	209	160	336	165	278	356	213	190	145	111	267	87	308	Silva, Jean Carlos Ramos, et al. “Cat-rodent Toxoplasma gondii Type II-variant circulation and limited genetic diversity on the Island of Fernando de Noronha, Brazil.” Parasites & vectors 10.1 (2017): 1-6.
BRA-TgCkBr213	Fernando de Noronha	Chicken (*Gallus domesticus*)	289	242	209	160	336	165	278	356	213	190	145	111	267	87	308	Silva, Jean Carlos Ramos, et al. “Cat-rodent Toxoplasma gondii Type II-variant circulation and limited genetic diversity on the Island of Fernando de Noronha, Brazil.” Parasites & vectors 10.1 (2017): 1-6.
BRA-TgCkBr214	Fernando de Noronha	Chicken (*Gallus domesticus*)	289	242	209	160	336	165	278	356	213	190	145	111	267	87	308	Silva, Jean Carlos Ramos, et al. “Cat-rodent Toxoplasma gondii Type II-variant circulation and limited genetic diversity on the Island of Fernando de Noronha, Brazil.” Parasites & vectors 10.1 (2017): 1-6.
BRA-TgCkBr215	Fernando de Noronha	Chicken (*Gallus domesticus*)	289	242	209	160	336	165	278	356	213	190	145	111	267	87	308	Silva, Jean Carlos Ramos, et al. “Cat-rodent Toxoplasma gondii Type II-variant circulation and limited genetic diversity on the Island of Fernando de Noronha, Brazil.” Parasites & vectors 10.1 (2017): 1-6.
BRA-TgCkBr216	Fernando de Noronha	Chicken (*Gallus domesticus*)	289	242	209	160	336	165	278	356	213	190	145	111	267	87	308	Silva, Jean Carlos Ramos, et al. “Cat-rodent Toxoplasma gondii Type II-variant circulation and limited genetic diversity on the Island of Fernando de Noronha, Brazil.” Parasites & vectors 10.1 (2017): 1-6.
BRA-TgCkBr217	Fernando de Noronha	Chicken (*Gallus domesticus*)	289	242	209	160	336	165	278	356	213	190	145	111	267	87	308	Silva, Jean Carlos Ramos, et al. “Cat-rodent Toxoplasma gondii Type II-variant circulation and limited genetic diversity on the Island of Fernando de Noronha, Brazil.” Parasites & vectors 10.1 (2017): 1-6.
BRA-TgCkBr218	Fernando de Noronha	Chicken (*Gallus domesticus*)	289	242	209	160	336	165	278	356	213	190	145	111	267	87	308	Silva, Jean Carlos Ramos, et al. “Cat-rodent Toxoplasma gondii Type II-variant circulation and limited genetic diversity on the Island of Fernando de Noronha, Brazil.” Parasites & vectors 10.1 (2017): 1-6.
BRA-TgCkBr219	Fernando de Noronha	Chicken (*Gallus domesticus*)	289	242	209	160	336	165	278	356	213	190	145	111	267	87	308	Silva, Jean Carlos Ramos, et al. “Cat-rodent Toxoplasma gondii Type II-variant circulation and limited genetic diversity on the Island of Fernando de Noronha, Brazil.” Parasites & vectors 10.1 (2017): 1-6.
BRA-TgCkBr220	Fernando de Noronha	Chicken (*Gallus domesticus*)	291	242	207	160	342	165	278	356	227	188	142	105	263	97	310	Silva, Jean Carlos Ramos, et al. “Cat-rodent Toxoplasma gondii Type II-variant circulation and limited genetic diversity on the Island of Fernando de Noronha, Brazil.” Parasites & vectors 10.1 (2017): 1-6.
BRA-TgCkBr221	Fernando de Noronha	Chicken (*Gallus domesticus*)	289	242	207	158	336	169	274	356	243	178	138	123	273	97	329	Silva, Jean Carlos Ramos, et al. “Cat-rodent Toxoplasma gondii Type II-variant circulation and limited genetic diversity on the Island of Fernando de Noronha, Brazil.” Parasites & vectors 10.1 (2017): 1-6.
BRA-TgCkBr222	Fernando de Noronha	Chicken (*Gallus domesticus*)	291	242	207	162	342	169	274	356	245	164	138	109	277	87	329	Silva, Jean Carlos Ramos, et al. “Cat-rodent Toxoplasma gondii Type II-variant circulation and limited genetic diversity on the Island of Fernando de Noronha, Brazil.” Parasites & vectors 10.1 (2017): 1-6.
BRA-TgCkBr223	Fernando de Noronha	Chicken (*Gallus domesticus*)	289	242	209	160	336	165	278	356	213	190	145	111	267	87	308	Silva, Jean Carlos Ramos, et al. “Cat-rodent Toxoplasma gondii Type II-variant circulation and limited genetic diversity on the Island of Fernando de Noronha, Brazil.” Parasites & vectors 10.1 (2017): 1-6.
BRA-TgCkBr224	Fernando de Noronha	Chicken (*Gallus domesticus*)	289	242	209	160	336	165	278	356	213	190	145	111	265	87	308	Silva, Jean Carlos Ramos, et al. “Cat-rodent Toxoplasma gondii Type II-variant circulation and limited genetic diversity on the Island of Fernando de Noronha, Brazil.” Parasites & vectors 10.1 (2017): 1-6.
BRA-TgCkBr225	Fernando de Noronha	Chicken (*Gallus domesticus*)	289	242	207	158	336	169	274	356	243	178	138	123	273	97	329	Silva, Jean Carlos Ramos, et al. “Cat-rodent Toxoplasma gondii Type II-variant circulation and limited genetic diversity on the Island of Fernando de Noronha, Brazil.” Parasites & vectors 10.1 (2017): 1-6.
BRA-TgCkBr226	Fernando de Noronha	Chicken (*Gallus domesticus*)	289	242	207	158	336	169	274	356	243	178	138	123	273	97	329	Silva, Jean Carlos Ramos, et al. “Cat-rodent Toxoplasma gondii Type II-variant circulation and limited genetic diversity on the Island of Fernando de Noronha, Brazil.” Parasites & vectors 10.1 (2017): 1-6.
BRA-TgCkBr227	Fernando de Noronha	Chicken (*Gallus domesticus*)	289	242	209	160	336	165	278	356	213	190	145	111	267	87	308	Silva, Jean Carlos Ramos, et al. “Cat-rodent Toxoplasma gondii Type II-variant circulation and limited genetic diversity on the Island of Fernando de Noronha, Brazil.” Parasites & vectors 10.1 (2017): 1-6.
BRA-TgCkBr228	Fernando de Noronha	Chicken (*Gallus domesticus*)	289	242	207	158	336	169	274	356	243	178	138	123	273	97	329	Silva, Jean Carlos Ramos, et al. “Cat-rodent Toxoplasma gondii Type II-variant circulation and limited genetic diversity on the Island of Fernando de Noronha, Brazil.” Parasites & vectors 10.1 (2017): 1-6.
BRA-TgCkBr229	Fernando de Noronha	Chicken (*Gallus domesticus*)	289	242	209	160	336	165	278	356	213	190	145	111	267	87	308	Silva, Jean Carlos Ramos, et al. “Cat-rodent Toxoplasma gondii Type II-variant circulation and limited genetic diversity on the Island of Fernando de Noronha, Brazil.” Parasites & vectors 10.1 (2017): 1-6.
BRA-TgCkBr230	Fernando de Noronha	Chicken (*Gallus domesticus*)	289	242	207	158	336	169	274	356	243	178	138	123	273	97	329	Silva, Jean Carlos Ramos, et al. “Cat-rodent Toxoplasma gondii Type II-variant circulation and limited genetic diversity on the Island of Fernando de Noronha, Brazil.” Parasites & vectors 10.1 (2017): 1-6.
BRA-TgCkBr231	Fernando de Noronha	Chicken (*Gallus domesticus*)	289	242	205	160	336	165	278	356	213	188	153	111	267	87	312	Silva, Jean Carlos Ramos, et al. “Cat-rodent Toxoplasma gondii Type II-variant circulation and limited genetic diversity on the Island of Fernando de Noronha, Brazil.” Parasites & vectors 10.1 (2017): 1-6.
BRA-TgCkBr232	Fernando de Noronha	Chicken (*Gallus domesticus*)	289	242	209	160	336	165	278	356	213	190	145	111	267	87	308	Silva, Jean Carlos Ramos, et al. “Cat-rodent Toxoplasma gondii Type II-variant circulation and limited genetic diversity on the Island of Fernando de Noronha, Brazil.” Parasites & vectors 10.1 (2017): 1-6.
BRA-TgCkBrSC1	Santa Catarina	Chicken (*Gallus domesticus*)	291	248	209	160	334	167	274	358	223	166	145	127	273	87	306	Pena, Hilda Fátima Jesus, et al. “Free-range chickens from Santa Catarina state, southern Brazil, as asymptomatic intermediate hosts for Toxoplasma gondii clonal type I and typical Brazilian genotypes.” *Veterinary Parasitology: Regional Studies and Reports* 13 (2018): 55-59.
BRA-TgCkBrSC2	Santa Catarina	Chicken (*Gallus domesticus*)	289	248	209	160	336	165	278	356	211	190	147	111	267	89	306	Pena, Hilda Fátima Jesus, et al. “Free-range chickens from Santa Catarina state, southern Brazil, as asymptomatic intermediate hosts for Toxoplasma gondii clonal type I and typical Brazilian genotypes.” *Veterinary Parasitology: Regional Studies and Reports* 13 (2018): 55-59.

			Microsatellite marker															
	Origin	Species	*TUB2*	*W35*	*TgM-A*	*B18*	*B17*	*M33*	*MIV.1*	*MXI.1*	*M48*	*M102*	*N60*	*N82*	*AA*	*N61*	*N83*	References
BRA-TgCkBrSC3	Santa Catarina	Chicken (*Gallus domesticus*)	289	242	205	162	342	165	278	356	237	164	145	111	295	93	341	Pena, Hilda Fátima Jesus, et al. "Free-range chickens from Santa Catarina state, southern Brazil, as asymptomatic intermediate hosts for Toxoplasma gondii clonal type I and typical Brazilian genotypes." *Veterinary Parasitology: Regional Studies and Reports* 13 (2018): 55-59.
BRA-TgCkBrSC4	Santa Catarina	Chicken (*Gallus domesticus*)	289	248	205	160	336	165	278	356	213	166	147	109	265	87	306	Pena, Hilda Fátima Jesus, et al. "Free-range chickens from Santa Catarina state, southern Brazil, as asymptomatic intermediate hosts for Toxoplasma gondii clonal type I and typical Brazilian genotypes." *Veterinary Parasitology: Regional Studies and Reports* 13 (2018): 55-59.
BRA-TgCpBr17	São Paulo	Capybara (*Hidrochoeris hidrochoeris*)	289	242	205	160	348	165	278	356	213	190	142	111	263	107	312	Pena, Hilda Fátima Jesus, et al. "First isolation and genotyping of Toxoplasma gondii in a free-living giant anteater (Myrmecophaga tridactyla) revealed a unique non-archetypal genotype." Acta Tropica 204 (2020): 105335.
BRA-TgCpBr18	São Paulo	Capybara (*Hidrochoeris hidrochoeris*)	289	242	205	160	348	165	278	356	213	190	142	111	261	127	310	Pena, Hilda Fátima Jesus, et al. "First isolation and genotyping of Toxoplasma gondii in a free-living giant anteater (Myrmecophaga tridactyla) revealed a unique non-archetypal genotype." Acta Tropica 204 (2020): 105335.
BRA-TgCpBr20	São Paulo	Capybara (*Hidrochoeris hidrochoeris*)	289	242	205	160	348	165	278	356	213	190	142	111	263	123	310	Pena, Hilda Fátima Jesus, et al. "First isolation and genotyping of Toxoplasma gondii in a free-living giant anteater (Myrmecophaga tridactyla) revealed a unique non-archetypal genotype." Acta Tropica 204 (2020): 105335.
BRA-TgCpBr36	São Paulo	Capybara (*Hidrochoeris hidrochoeris*)	289	242	205	160	348	165	278	356	213	190	142	111	263	107	312	Pena, Hilda Fátima Jesus, et al. "First isolation and genotyping of Toxoplasma gondii in a free-living giant anteater (Myrmecophaga tridactyla) revealed a unique non-archetypal genotype." Acta Tropica 204 (2020): 105335.
BRA-TgDgBr06	São Paulo	Dog (*Canis familiaris*)	291	248	205	160	362	165	278	354	231	166	151	111	273	91	343	Su, Chunlei, et al. "Globally diverse Toxoplasma gondii isolates comprise six major clades originating from a small number of distinct ancestral lineages." Proceedings of the National Academy of Sciences 109.15 (2012): 5844-5849.
BRA-TgDgBr15	São Paulo	Dog (*Canis familiaris*)	291	242	205	162	362	165	278	356	233	166	149	111	295	91	341	Su, Chunlei, et al. "Globally diverse Toxoplasma gondii isolates comprise six major clades originating from a small number of distinct ancestral lineages." Proceedings of the National Academy of Sciences 109.15 (2012): 5844-5849.
BRA-TgDgBr18	São Paulo	Dog (*Canis familiaris*)	291	242	205	162	338	169	276	358	229	164	142	111	324	89	316	Su, Chunlei, et al. "Globally diverse Toxoplasma gondii isolates comprise six major clades originating from a small number of distinct ancestral lineages." Proceedings of the National Academy of Sciences 109.15 (2012): 5844-5849.
BRA-TgGtBr10	Northeast Brazil	Goat (*Capra aegagrus hircus*)	289	242	205	160	338	165	274	356	237	166	145	107	312	99	314	Ragozo AM et al. Genetic diversity among Toxoplasma gondii isolates of small ruminants from Brazil: novel genotypes revealed. Vet Parasitol. 2010 Jun 24;170(3-4):307-12.
BRA-TgRatno-BrFN1	Fernando de Noronha	Brown rat (*Rattus norvegicus*)	289	242	207	158	336	169	274	356	235	178	138	123	271	97	329	Silva, Jean Carlos Ramos, et al. "Cat-rodent Toxoplasma gondii Type II-variant circulation and limited genetic diversity on the Island of Fernando de Noronha, Brazil." Parasites & vectors 10.1 (2017): 1-6.
BRA-TgRatno-BrFN2	Fernando de Noronha	Brown rat (*Rattus norvegicus*)	289	242	209	160	336	165	278	356	213	190	145	111	267	87	308	Silva, Jean Carlos Ramos, et al. "Cat-rodent Toxoplasma gondii Type II-variant circulation and limited genetic diversity on the Island of Fernando de Noronha, Brazil." Parasites & vectors 10.1 (2017): 1-6.
BRA-TgRatRat01	Fernando de Noronha	Black rat (*Rattus rattus*)	291	242	205	162	342	165	278	356	213	164	142	109	279	87	312	Silva, Jean Carlos Ramos, et al. "Cat-rodent Toxoplasma gondii Type II-variant circulation and limited genetic diversity on the Island of Fernando de Noronha, Brazil." Parasites & vectors 10.1 (2017): 1-6.
LPN-MAS	unknown	Human (*Homo sapiens*)	291	242	205	162	362	169	272	358	221	166	142	111	332	95	339	Su, Chunlei, et al. "Globally diverse Toxoplasma gondii isolates comprise six major clades originating from a small number of distinct ancestral lineages." Proceedings of the National Academy of Sciences 109.15 (2012): 5844-5849.
PS-TgCatBrSC1	Santa Catarina	Cat (*Felis catis*)	291	248	209	160	334	167	274	358	223	166	145	129	269	87	306	Pena, Hilda Fátima de Jesus, et al. "Fatal toxoplasmosis in an immunosuppressed domestic cat from Brazil caused by Toxoplasma gondii clonal type I." Revista Brasileira de Parasitologia Veterinária 26.2 (2017): 177-184.
PS-TgCatEgBr1	Fernando de Noronha	Cattle Egret (*Bubulcus ibis*)	289	242	209	160	336	165	278	356	213	190	145	111	265	87	308	Silva, Jean Carlos Ramos, et al. "Cat-rodent Toxoplasma gondii Type II-variant circulation and limited genetic diversity on the Island of Fernando de Noronha, Brazil." Parasites & vectors 10.1 (2017): 1-6.
PS-TgCatEgBr2	Fernando de Noronha	Cattle Egret (*Bubulcus ibis*)	289	242	209	160	336	165	278	356	213	190	145	111	267	87	308	Silva, Jean Carlos Ramos, et al. "Cat-rodent Toxoplasma gondii Type II-variant circulation and limited genetic diversity on the Island of Fernando de Noronha, Brazil." Parasites & vectors 10.1 (2017): 1-6.
PS-TgPigBrPB1	Paraiba	Pig (*Sus scrofa domesticus*)	293	242	211	160	336	169	274	354	219	172	130	111	277	99	308	Olinda, Roberio G., et al. "Acute toxoplasmosis in pigs in Brazil caused by Toxoplasma gondii genotype Chinese 1." Parasitology research 115.7 (2016): 2561-2566.
PS-TgPigBrPB2	Paraiba	Pig (*Sus scrofa domesticus*)	293	242	211	160	336	169	274	354	219	172	130	111	277	99	308	Olinda, Roberio G., et al. "Acute toxoplasmosis in pigs in Brazil caused by Toxoplasma gondii genotype Chinese 1." Parasitology research 115.7 (2016): 2561-2566.
RsTgOvBr2	São Paulo	Sheep (*Ovis aries*)	289	242	207	158	336	169	274	356	213	176	140	111	261	89	314	da Silva RA et al. Genotypic characterization of Toxoplasma gondii in sheep from Brazilian slaughterhouses: New atypical genotypes and the clonal type II strain identified. Vet. Parasitol. 2011, 175:173–177.
RsTgOvBr5	São Paulo	Sheep (*Ovis aries*)	289	242	207	158	360	169	274	ND	215	174	140	111	263	91	312	da Silva RA et al. Genotypic characterization of Toxoplasma gondii in sheep from Brazilian slaughterhouses: New atypical genotypes and the clonal type II strain identified. Vet. Parasitol. 2011, 175:173–177.
TgCkBrRS20	Rio Grande do Sul	Chicken (*Gallus domesticus*)	291	248	209	160	348	165	278	356	213	166	140	111	265	87	306	Vielmo, Andréia, et al. "Outbreak of toxoplasmosis in a flock of domestic chickens (Gallus gallus domesticus) and guinea fowl (Numida meleagris)." *Parasitology research* 118.3 (2019): 991-997.
TgCkBrRS21	Rio Grande do Sul	Chicken (*Gallus domesticus*)	291	248	209	160	348	165	278	356	213	166	140	111	265	87	306	Vielmo, Andréia, et al. "Outbreak of toxoplasmosis in a flock of domestic chickens (Gallus gallus domesticus) and guinea fowl (Numida meleagris)." *Parasitology research* 118.3 (2019): 991-997.
TgJagBr1	Northeast Brazil	Jaguarundi (*Puma yagouaroundi*)	289	242	205	162	344	165	278	358	235	164	138	111	263	91	312	Pena et al. Isolation and genetic characterisation of Toxoplasma gondii from a red-handed howler monkey (Alouatta belzebul), a jaguarundi (Puma yagouaroundi), and a black-eared opossum (Didelphis aurita) from Brazil. Vet Parasitol. 2011 Feb 10;175(3-4):377-81
TgOncBr1	Northeast Brazil	Oncilla (*Leopardus tigrinus*)	291	242	205	160	342	165	278	356	227	166	147	111	281	93	308	Vitaliano SN et al. Genetic characterization of Toxoplasma gondii from Brazilian wildlife revealed abundant new genotypes. Int J Parasitol Parasites Wildl. 2014 Oct 13;3(3):276-83.

			Microsatellite marker															
	Origin	Species	*TUB2*	*W35*	*TgM-A*	*B18*	*B17*	*M33*	*MIV.1*	*MXI.1*	*M48*	*M102*	*N60*	*N82*	*AA*	*N61*	*N83*	References
TgRhHmBr1	Northeast Brazil	Red-handed howler monkey (*Alouatta belzebul*)	291	242	205	162	342	165	278	356	213	164	142	109	277	87	312	Pena et al. Isolation and genetic characterisation of Toxoplasma gondii from a red-handed howler monkey (Alouatta belzebul), a jaguarundi (Puma yagouaroundi), and a black-eared opossum (Didelphis aurita) from Brazil. Vet Parasitol. 2011 Feb 10;175(3-4):377-81
PS-TgRabbit-BrRS1	Rio Grande do Sul	Rabbit (*Oryctolagus cuniculus*)	291	242	205	159	348	165	278	355	213	188	142	111	261	89	314	Pena, Hilda Fátima Jesus, et al. “First isolation and genotyping of Toxoplasma gondii in a free-living giant anteater (Myrmecophaga tridactyla) revealed a unique non-archetypal genotype.” Acta Tropica 204 (2020): 105335.
TgPigBrPB19	Paraiba	Pig (*Sus scrofa domesticus*)	289	242	205	160	336	165	278	356	211	190	138	111	271	97	304	Feitosa, Thais Ferreira, et al. “High genetic diversity in Toxoplasma gondii isolates from pigs at slaughterhouses in Paraíba state, northeastern Brazil: circulation of new genotypes and Brazilian clonal lineages.” Veterinary parasitology 244 (2017): 76-80.
TgPigBrPB23	Paraiba	Pig (*Sus scrofa domesticus*)	289	242	205	160	336	165	278	356	227	190	142	105	269	103	306	Feitosa, Thais Ferreira, et al. “High genetic diversity in Toxoplasma gondii isolates from pigs at slaughterhouses in Paraíba state, northeastern Brazil: circulation of new genotypes and Brazilian clonal lineages.” Veterinary parasitology 244 (2017): 76-80.
TgPigBrPB17	Paraiba	Pig (*Sus scrofa domesticus*)	291	242	205	162	342	165	278	356	213	164	142	109	275	87	312	Feitosa, Thais Ferreira, et al. “High genetic diversity in Toxoplasma gondii isolates from pigs at slaughterhouses in Paraíba state, northeastern Brazil: circulation of new genotypes and Brazilian clonal lineages.” Veterinary parasitology 244 (2017): 76-80.
TgPigBrPB18	Paraiba	Pig (*Sus scrofa domesticus*)	291	242	205	162	342	165	278	356	217	164	142	109	277	87	312	Feitosa, Thais Ferreira, et al. “High genetic diversity in Toxoplasma gondii isolates from pigs at slaughterhouses in Paraíba state, northeastern Brazil: circulation of new genotypes and Brazilian clonal lineages.” Veterinary parasitology 244 (2017): 76-80.
TgPigBrPB20	Paraiba	Pig (*Sus scrofa domesticus*)	291	242	205	162	342	165	278	356	213	164	142	109	277	87	312	Feitosa, Thais Ferreira, et al. “High genetic diversity in Toxoplasma gondii isolates from pigs at slaughterhouses in Paraíba state, northeastern Brazil: circulation of new genotypes and Brazilian clonal lineages.” Veterinary parasitology 244 (2017): 76-80.
TgPigBrPB27	Paraiba	Pig (*Sus scrofa domesticus*)	291	242	205	162	342	165	278	356	213	164	142	109	277	87	312	Feitosa, Thais Ferreira, et al. “High genetic diversity in Toxoplasma gondii isolates from pigs at slaughterhouses in Paraíba state, northeastern Brazil: circulation of new genotypes and Brazilian clonal lineages.” Veterinary parasitology 244 (2017): 76-80.
TgPigBrPB13	Paraiba	Pig (*Sus scrofa domesticus*)	291	242	205	162	342	165	278	356	213	164	142	105	279	89	312	Feitosa, Thais Ferreira, et al. “High genetic diversity in Toxoplasma gondii isolates from pigs at slaughterhouses in Paraíba state, northeastern Brazil: circulation of new genotypes and Brazilian clonal lineages.” Veterinary parasitology 244 (2017): 76-80.
TgPigBrPB8	Paraiba	Pig (*Sus scrofa domesticus*)	291	242	205	160	342	165	278	356	233	166	140	105	263	95	306	Feitosa, Thais Ferreira, et al. “High genetic diversity in Toxoplasma gondii isolates from pigs at slaughterhouses in Paraíba state, northeastern Brazil: circulation of new genotypes and Brazilian clonal lineages.” Veterinary parasitology 244 (2017): 76-80.
TgPigBrPB24	Paraiba	Pig (*Sus scrofa domesticus*)	291	242	207	166	334	165	278	356	225	168	147	105	293	91	310	Feitosa, Thais Ferreira, et al. “High genetic diversity in Toxoplasma gondii isolates from pigs at slaughterhouses in Paraíba state, northeastern Brazil: circulation of new genotypes and Brazilian clonal lineages.” Veterinary parasitology 244 (2017): 76-80.
TgPigBrPB16	Paraiba	Pig (*Sus scrofa domesticus*)	289	242	207	160	342	165	278	356	211	190	145	105	265	113	306	Feitosa, Thais Ferreira, et al. “High genetic diversity in Toxoplasma gondii isolates from pigs at slaughterhouses in Paraíba state, northeastern Brazil: circulation of new genotypes and Brazilian clonal lineages.” Veterinary parasitology 244 (2017): 76-80.
TgPigBrPB11	Paraiba	Pig (*Sus scrofa domesticus*)	291	242	205	162	356	165	278	356	213	164	140	111	265	121	312	Feitosa, Thais Ferreira, et al. “High genetic diversity in Toxoplasma gondii isolates from pigs at slaughterhouses in Paraíba state, northeastern Brazil: circulation of new genotypes and Brazilian clonal lineages.” Veterinary parasitology 244 (2017): 76-80.
TgPigBrPB14	Paraiba	Pig (*Sus scrofa domesticus*)	289	242	205	160	348	169	278	356	215	196	140	111	265	117	312	Feitosa, Thais Ferreira, et al. “High genetic diversity in Toxoplasma gondii isolates from pigs at slaughterhouses in Paraíba state, northeastern Brazil: circulation of new genotypes and Brazilian clonal lineages.” Veterinary parasitology 244 (2017): 76-80.
TgPigBrPB3	Paraiba	Pig (*Sus scrofa domesticus*)	291	248	205	160	336	165	278	356	231	166	138	105	263	89	312	Feitosa, Thais Ferreira, et al. “High genetic diversity in Toxoplasma gondii isolates from pigs at slaughterhouses in Paraíba state, northeastern Brazil: circulation of new genotypes and Brazilian clonal lineages.” Veterinary parasitology 244 (2017): 76-80.
TgPigBrPB26	Paraiba	Pig (*Sus scrofa domesticus*)	291	248	205	160	336	165	278	356	231	166	138	105	263	89	312	Feitosa, Thais Ferreira, et al. “High genetic diversity in Toxoplasma gondii isolates from pigs at slaughterhouses in Paraíba state, northeastern Brazil: circulation of new genotypes and Brazilian clonal lineages.” Veterinary parasitology 244 (2017): 76-80.
TgPigBrPB9	Paraiba	Pig (*Sus scrofa domesticus*)	291	242	207	162	342	169	278	356	213	164	147	111	265	105	312	Feitosa, Thais Ferreira, et al. “High genetic diversity in Toxoplasma gondii isolates from pigs at slaughterhouses in Paraíba state, northeastern Brazil: circulation of new genotypes and Brazilian clonal lineages.” Veterinary parasitology 244 (2017): 76-80.
TgPigBrPB21	Paraiba	Pig (*Sus scrofa domesticus*)	291	242	207	162	342	169	278	356	213	164	147	111	265	95	312	Feitosa, Thais Ferreira, et al. “High genetic diversity in Toxoplasma gondii isolates from pigs at slaughterhouses in Paraíba state, northeastern Brazil: circulation of new genotypes and Brazilian clonal lineages.” Veterinary parasitology 244 (2017): 76-80.
TgPigBrPB10	Paraiba	Pig (*Sus scrofa domesticus*)	289	242	205	160	336	167	274	356	211	190	142	123	267	93	304	Feitosa, Thais Ferreira, et al. “High genetic diversity in Toxoplasma gondii isolates from pigs at slaughterhouses in Paraíba state, northeastern Brazil: circulation of new genotypes and Brazilian clonal lineages.” Veterinary parasitology 244 (2017): 76-80.
TgPigBrPB12	Paraiba	Pig (*Sus scrofa domesticus*)	291	248	205	160	342	165	278	356	235	166	151	105	269	93	306	Feitosa, Thais Ferreira, et al. “High genetic diversity in Toxoplasma gondii isolates from pigs at slaughterhouses in Paraíba state, northeastern Brazil: circulation of new genotypes and Brazilian clonal lineages.” Veterinary parasitology 244 (2017): 76-80.
TgCkBrMA1	Maranhao	Chicken (*Gallus domesticus*)	291	242	207	166	334	165	278	356	239	168	147	105	289	93	310	Sousa, I. C., et al. “First isolation and genotyping of Toxoplasma gondii from free-range chickens on Sao Luis island, Maranhao state, Brazil, with a new genotype described.” Veterinary parasitology 223 (2016): 159-164.
TgCkBrMA2	Maranhao	Chicken (*Gallus domesticus*)	289	242	203	156	340	165	278	356	241	170	142	111	271	95	312	Sousa, I. C., et al. “First isolation and genotyping of Toxoplasma gondii from free-range chickens on Sao Luis island, Maranhao state, Brazil, with a new genotype described.” Veterinary parasitology 223 (2016): 159-164.
TgCkBrMA3	Maranhao	Chicken (*Gallus domesticus*)	289	242	203	156	340	165	278	356	223	170	142	111	269	93	312	Sousa, I. C., et al. “First isolation and genotyping of Toxoplasma gondii from free-range chickens on Sao Luis island, Maranhao state, Brazil, with a new genotype described.” Veterinary parasitology 223 (2016): 159-164.
TgCkBrMA4	Maranhao	Chicken (*Gallus domesticus*)	291	248	205	160	342	165	274	354	223	166	147	111	275	89	306	Sousa, I. C., et al. “First isolation and genotyping of Toxoplasma gondii from free-range chickens on Sao Luis island, Maranhao state, Brazil, with a new genotype described.” Veterinary parasitology 223 (2016): 159-164.

			Microsatellite marker															
	Origin	Species	*TUB2*	*W35*	*TgM-A*	*B18*	*B17*	*M33*	*MIV.1*	*MXI.1*	*M48*	*M102*	*N60*	*N82*	*AA*	*N61*	*N83*	References
TgCkBrMA5	Maranhao	Chicken (*Gallus domesticus*)	289	248	205	162	342	165	274	356	213	164	142	111	263	89	314	Sousa, I. C., et al. "First isolation and genotyping of Toxoplasma gondii from free-range chickens on Sao Luis island, Maranhao state, Brazil, with a new genotype described." Veterinary parasitology 223 (2016): 159-164.
TgHorseBrRS1	Rio Grande do Sul	Horse (*Equus ferus caballus*)	291	242	207	158	358	169	274	356	225	164	153	109	263	97	310	Pena, Hilda Fátima Jesus, et al. "Typical Brazilian genotype of Toxoplasma gondii isolated from a horse destined for human consumption in Europe from a slaughterhouse." Parasitology research 117.10 (2018): 3305-3308.
PS-TgLonloBrPE1	Pernambuco	Otter (*Lontra longicaudis*)	291	242	205	162	342	165	278	356	213	164	142	109	277	87	312	Silva, Marcio André, et al. "Isolation and genetic characterization of Toxoplasma gondii from free-ranging and captive birds and mammals in Pernambuco state, Brazil." Revista Brasileira de Parasitologia Veterinária 27.4 (2018): 481-487.
PS-TgBraarBrSP1	São Paulo	Southern muriqui (*Brachyteles arachnoides*)	291	242	207	160	360	165	278	356	241	176	140	105	263	91	314	Santos SV et al. Fatal toxoplasmosis in a southern muriqui (Brachyteles arachnoides) from São Paulo state, Brazil: Pathological, immunohistochemical, and molecular characterization. J Med Primatol. 2018 Apr;47(2):124-127.
TgCkBrPB3	Paraiba	Chicken (*Gallus domesticus*)	291	242	205	162	342	165	278	356	213	164	142	109	277	87	312	Feitosa, Thais Ferreira, et al. "First report of typical Brazilian Toxoplasma gondii genotypes from isolates of free-range chickens (Gallus gallus domesticus) circulating in the state of Paraíba, Northeast Brazil." Parasitology research 116.8 (2017): 2265-2270.
TgCkBrPB10	Paraiba	Chicken (*Gallus domesticus*)	291	242	205	162	342	165	278	356	215	164	142	109	277	87	312	Feitosa, Thais Ferreira, et al. "First report of typical Brazilian Toxoplasma gondii genotypes from isolates of free-range chickens (Gallus gallus domesticus) circulating in the state of Paraíba, Northeast Brazil." Parasitology research 116.8 (2017): 2265-2270.
TgCkBrPB13	Paraiba	Chicken (*Gallus domesticus*)	291	242	205	162	342	165	278	356	215	164	142	109	277	87	312	Feitosa, Thais Ferreira, et al. "First report of typical Brazilian Toxoplasma gondii genotypes from isolates of free-range chickens (Gallus gallus domesticus) circulating in the state of Paraíba, Northeast Brazil." Parasitology research 116.8 (2017): 2265-2270.
TgCkBrPB15	Paraiba	Chicken (*Gallus domesticus*)	291	242	205	162	342	165	278	356	213	164	142	109	277	85	312	Feitosa, Thais Ferreira, et al. "First report of typical Brazilian Toxoplasma gondii genotypes from isolates of free-range chickens (Gallus gallus domesticus) circulating in the state of Paraíba, Northeast Brazil." Parasitology research 116.8 (2017): 2265-2270.
TgCkBrPB16	Paraiba	Chicken (*Gallus domesticus*)	291	242	205	162	342	165	278	356	213	164	142	109	277	85	312	Feitosa, Thais Ferreira, et al. "First report of typical Brazilian Toxoplasma gondii genotypes from isolates of free-range chickens (Gallus gallus domesticus) circulating in the state of Paraíba, Northeast Brazil." Parasitology research 116.8 (2017): 2265-2270.
TgCkBrPB17	Paraiba	Chicken (*Gallus domesticus*)	291	242	205	162	342	165	278	356	213	164	142	109	277	89	312	Feitosa, Thais Ferreira, et al. "First report of typical Brazilian Toxoplasma gondii genotypes from isolates of free-range chickens (Gallus gallus domesticus) circulating in the state of Paraíba, Northeast Brazil." Parasitology research 116.8 (2017): 2265-2270.
TgCkBrPB18	Paraiba	Chicken (*Gallus domesticus*)	291	242	205	162	342	165	278	356	215	164	142	109	277	87	312	Feitosa, Thais Ferreira, et al. "First report of typical Brazilian Toxoplasma gondii genotypes from isolates of free-range chickens (Gallus gallus domesticus) circulating in the state of Paraíba, Northeast Brazil." Parasitology research 116.8 (2017): 2265-2270.
TgCkBrPB19	Paraiba	Chicken (*Gallus domesticus*)	291	242	205	162	342	165	278	356	213	164	142	109	277	87	312	Feitosa, Thais Ferreira, et al. "First report of typical Brazilian Toxoplasma gondii genotypes from isolates of free-range chickens (Gallus gallus domesticus) circulating in the state of Paraíba, Northeast Brazil." Parasitology research 116.8 (2017): 2265-2270.
TgCkBrPB20	Paraiba	Chicken (*Gallus domesticus*)	291	242	205	162	342	165	278	356	213	164	142	109	277	87	312	Feitosa, Thais Ferreira, et al. "First report of typical Brazilian Toxoplasma gondii genotypes from isolates of free-range chickens (Gallus gallus domesticus) circulating in the state of Paraíba, Northeast Brazil." Parasitology research 116.8 (2017): 2265-2270.
TgCkBrPB22	Paraiba	Chicken (*Gallus domesticus*)	291	242	205	162	342	165	278	356	213	164	142	109	277	89	312	Feitosa, Thais Ferreira, et al. "First report of typical Brazilian Toxoplasma gondii genotypes from isolates of free-range chickens (Gallus gallus domesticus) circulating in the state of Paraíba, Northeast Brazil." Parasitology research 116.8 (2017): 2265-2270.
TgCkBrPB23	Paraiba	Chicken (*Gallus domesticus*)	291	242	205	162	342	165	278	356	213	164	142	109	277	89	312	Feitosa, Thais Ferreira, et al. "First report of typical Brazilian Toxoplasma gondii genotypes from isolates of free-range chickens (Gallus gallus domesticus) circulating in the state of Paraíba, Northeast Brazil." Parasitology research 116.8 (2017): 2265-2270.
TgCkBrPB24	Paraiba	Chicken (*Gallus domesticus*)	291	242	205	162	342	165	278	356	213	164	142	109	277	89	312	Feitosa, Thais Ferreira, et al. "First report of typical Brazilian Toxoplasma gondii genotypes from isolates of free-range chickens (Gallus gallus domesticus) circulating in the state of Paraíba, Northeast Brazil." Parasitology research 116.8 (2017): 2265-2270.
TgCkBrPB25	Paraiba	Chicken (*Gallus domesticus*)	291	242	205	162	342	165	278	356	213	164	142	109	277	87	312	Feitosa, Thais Ferreira, et al. "First report of typical Brazilian Toxoplasma gondii genotypes from isolates of free-range chickens (Gallus gallus domesticus) circulating in the state of Paraíba, Northeast Brazil." Parasitology research 116.8 (2017): 2265-2270.
TgCkBrPB27	Paraiba	Chicken (*Gallus domesticus*)	291	242	205	162	342	165	278	356	213	164	142	109	277	89	312	Feitosa, Thais Ferreira, et al. "First report of typical Brazilian Toxoplasma gondii genotypes from isolates of free-range chickens (Gallus gallus domesticus) circulating in the state of Paraíba, Northeast Brazil." Parasitology research 116.8 (2017): 2265-2270.
TgCkBrPB9	Paraiba	Chicken (*Gallus domesticus*)	289	242	205	160	342	165	278	358	235	164	145	111	316	87	308	Feitosa, Thais Ferreira, et al. "First report of typical Brazilian Toxoplasma gondii genotypes from isolates of free-range chickens (Gallus gallus domesticus) circulating in the state of Paraíba, Northeast Brazil." Parasitology research 116.8 (2017): 2265-2270.
TgCkBrPB30	Paraiba	Chicken (*Gallus domesticus*)	289	242	205	160	336	165	278	356	211	190	138	111	275	97	304	Feitosa, Thais Ferreira, et al. "First report of typical Brazilian Toxoplasma gondii genotypes from isolates of free-range chickens (Gallus gallus domesticus) circulating in the state of Paraíba, Northeast Brazil." Parasitology research 116.8 (2017): 2265-2270.
TgCkBrPB4	Paraiba	Chicken (*Gallus domesticus*)	289	242	205	162	336	165	278	358	219	172	140	111	265	101	304	Feitosa, Thais Ferreira, et al. "First report of typical Brazilian Toxoplasma gondii genotypes from isolates of free-range chickens (Gallus gallus domesticus) circulating in the state of Paraíba, Northeast Brazil." Parasitology research 116.8 (2017): 2265-2270.
TgCkBrPB5	Paraiba	Chicken (*Gallus domesticus*)	289	242	205	162	336	165	278	358	219	172	140	111	265	101	304	Feitosa, Thais Ferreira, et al. "First report of typical Brazilian Toxoplasma gondii genotypes from isolates of free-range chickens (Gallus gallus domesticus) circulating in the state of Paraíba, Northeast Brazil." Parasitology research 116.8 (2017): 2265-2270.
TgCkBrPB6	Paraiba	Chicken (*Gallus domesticus*)	289	242	205	162	336	165	278	358	219	172	140	111	265	101	304	Feitosa, Thais Ferreira, et al. "First report of typical Brazilian Toxoplasma gondii genotypes from isolates of free-range chickens (Gallus gallus domesticus) circulating in the state of Paraíba, Northeast Brazil." Parasitology research 116.8 (2017): 2265-2270.
TgCkBrPB28	Paraiba	Chicken (*Gallus domesticus*)	291	248	205	160	342	165	278	354	229	166	142	105	267	91	306	Feitosa, Thais Ferreira, et al. "First report of typical Brazilian Toxoplasma gondii genotypes from isolates of free-range chickens (Gallus gallus domesticus) circulating in the state of Paraíba, Northeast Brazil." Parasitology research 116.8 (2017): 2265-2270.

	Origin	Species	Microsatellite marker															References
			TUB2	*W35*	*TgM-A*	*B18*	*B17*	*M33*	*MIV.1*	*MXI.1*	*M48*	*M102*	*N60*	*N82*	*AA*	*N61*	*N83*	
TgCkBrPB29	Paraiba	Chicken (*Gallus domesticus*)	291	248	205	160	342	165	278	354	227	166	142	105	263	89	306	Feitosa, Thais Ferreira, et al. "First report of typical Brazilian Toxoplasma gondii genotypes from isolates of free-range chickens (Gallus gallus domesticus) circulating in the state of Paraíba, Northeast Brazil." Parasitology research 116.8 (2017): 2265-2270.
TgCkBrPB1	Paraiba	Chicken (*Gallus domesticus*)	289	242	205	160	348	169	278	356	215	196	140	111	265	119	312	Feitosa, Thais Ferreira, et al. "First report of typical Brazilian Toxoplasma gondii genotypes from isolates of free-range chickens (Gallus gallus domesticus) circulating in the state of Paraíba, Northeast Brazil." Parasitology research 116.8 (2017): 2265-2270.
TgCkBrPB2	Paraiba	Chicken (*Gallus domesticus*)	289	242	205	160	348	169	278	356	215	196	140	111	265	119	312	Feitosa, Thais Ferreira, et al. "First report of typical Brazilian Toxoplasma gondii genotypes from isolates of free-range chickens (Gallus gallus domesticus) circulating in the state of Paraíba, Northeast Brazil." Parasitology research 116.8 (2017): 2265-2270.
TgCkBrPB11	Paraiba	Chicken (*Gallus domesticus*)	291	242	207	162	342	169	278	356	213	164	147	111	265	97	312	Feitosa, Thais Ferreira, et al. "First report of typical Brazilian Toxoplasma gondii genotypes from isolates of free-range chickens (Gallus gallus domesticus) circulating in the state of Paraíba, Northeast Brazil." Parasitology research 116.8 (2017): 2265-2270.
TgCkBrPB12	Paraiba	Chicken (*Gallus domesticus*)	291	242	207	162	342	169	278	356	213	164	147	111	265	95	312	Feitosa, Thais Ferreira, et al. "First report of typical Brazilian Toxoplasma gondii genotypes from isolates of free-range chickens (Gallus gallus domesticus) circulating in the state of Paraíba, Northeast Brazil." Parasitology research 116.8 (2017): 2265-2270.
TgCkBrPB14	Paraiba	Chicken (*Gallus domesticus*)	291	242	207	162	342	169	278	356	213	164	147	111	265	99	312	Feitosa, Thais Ferreira, et al. "First report of typical Brazilian Toxoplasma gondii genotypes from isolates of free-range chickens (Gallus gallus domesticus) circulating in the state of Paraíba, Northeast Brazil." Parasitology research 116.8 (2017): 2265-2270.
TgCkBrPB26	Paraiba	Chicken (*Gallus domesticus*)	291	242	205	160	342	165	278	356	233	166	140	105	263	95	306	Feitosa, Thais Ferreira, et al. "First report of typical Brazilian Toxoplasma gondii genotypes from isolates of free-range chickens (Gallus gallus domesticus) circulating in the state of Paraíba, Northeast Brazil." Parasitology research 116.8 (2017): 2265-2270.
TgCkBrPB7	Paraiba	Chicken (*Gallus domesticus*)	289	242	205	160	336	165	278	356	225	190	142	105	275	99	304	Feitosa, Thais Ferreira, et al. "First report of typical Brazilian Toxoplasma gondii genotypes from isolates of free-range chickens (Gallus gallus domesticus) circulating in the state of Paraíba, Northeast Brazil." Parasitology research 116.8 (2017): 2265-2270.
TgCkBrPB8	Paraiba	Chicken (*Gallus domesticus*)	289	242	205	160	336	165	278	356	225	190	142	105	275	99	304	Feitosa, Thais Ferreira, et al. "First report of typical Brazilian Toxoplasma gondii genotypes from isolates of free-range chickens (Gallus gallus domesticus) circulating in the state of Paraíba, Northeast Brazil." Parasitology research 116.8 (2017): 2265-2270.
TgMytrBrSP1	São Paulo	Giant anteater (*Myrmecophaga tridactyla*)	289	242	205	160	348	165	278	356	213	190	142	111	263	103	312	Pena, Hilda Fátima Jesus, et al. "First isolation and genotyping of Toxoplasma gondii in a free-living giant anteater (Myrmecophaga tridactyla) revealed a unique non-archetypal genotype." *Acta tropica* 204 (2020): 105335.
Pumayag1-RJ	Rio de Janeiro	Jaguarondi (*Puma yagouaroundi*)	289	242	205	160	348	165	278	356	213	190	142	111	263	111	312	This study
